# *Scutellaria baicalensis* enhances 5-fluorouracil-based chemotherapy via inhibition of proliferative signaling pathways

**DOI:** 10.1186/s12964-023-01156-7

**Published:** 2023-06-19

**Authors:** Haizhou Liu, Hui Liu, Zhiyi Zhou, Jessica Chung, Guojing Zhang, Jin Chang, Robert A. Parise, Edward Chu, John C. Schmitz

**Affiliations:** 1 Division of Hematology-Oncology, Department of Medicine, University of Pittsburgh, Pittsburgh, PA USA; 2 Cancer Therapeutics Program, UPMC Hillman Cancer Center, University of Pittsburgh, 5117 Centre Ave, Pittsburgh, PA 15213 USA; 3 Department of Oncology, Putuo Hospital, Shanghai University of Traditional Chinese Medicine, Shanghai, China; 4 Department of Oncology, Longhua Hospital, Shanghai University of Traditional Chinese Medicine, Shanghai, China; 5 Department of Obstetrics and Gynecology, Abington-Jefferson Health, Abington, PA USA; 6 Department of Radiotherapy, Second Affiliated Hospital, Shandong First Medical University, Tai’an City, China; 7 Albert Einstein Cancer Center, Cancer Therapeutics Program, Albert Einstein College of Medicine, Bronx, NY USA

**Keywords:** Scutellaria baicalensis, Colorectal cancer, 5-fluorouracil, CDK-RB pathway, Baicalin

## Abstract

Fluoropyridine-based chemotherapy remains the most widely used treatment for colorectal cancer (CRC). In this study, we investigated the mechanism by which the natural product *Scutellaria baicalensis* (Huang Qin; HQ) and one of its main components baicalin enhanced 5-fluorouracil (5-FU) antitumor activity against CRC. Cell proliferation assays, cell cycle analysis, reverse-phase protein array (RPPA) analysis, immunoblot analysis, and qRT-PCR were performed to investigate the mechanism(s) of action of HQ and its active components on growth of CRC cells. HQ exhibited in vitro antiproliferative activity against drug resistant human CRC cells, against human and mouse CRC cells with different genetic backgrounds and normal human colon epithelial cells. In vivo animal models were used to document the antitumor activity of HQ and baicalin. The mechanism of growth inhibitory activity of HQ is due to inhibition of proliferative signaling pathways including the CDK-RB pathway. In addition, HQ enhanced the antitumor effects of 5-FU and capecitabine in vivo*.* Furthermore, we identified baicalin as an active component of HQ. The combination of baicalin and 5-FU demonstrated synergistic activity against 5-FU-resistant RKO-R10 cells. The combination significantly inhibited in vivo tumor growth greater than each treatment alone. RPPA results showed that the signaling pathway alterations in CRC cells were similar following HQ and baicalin treatment. Together, these results indicate that HQ and its component baicalin enhance the effect of 5-fluorouracil-based chemotherapy via inhibition of CDK-RB pathway. These findings may provide the rational basis for developing agents that can overcome the development of cellular drug resistance.

Video Abstract

**Supplementary Information:**

The online version contains supplementary material available at 10.1186/s12964-023-01156-7.

## Background

Colorectal cancer (CRC) is a major public health problem throughout the world and is the third most common cause of cancer death. In 2022, it is estimated that nearly 151,000 individuals will be diagnosed with CRC and nearly 53,000 will die from the disease [1]. Significant treatment advances have been developed with median survival rates in metastatic CRC patients now approaching 3 years [2]. However, current therapies remain limited in their overall clinical efficacy, and only a small minority of patients are able to achieve a real cure, as defined by 5-year survival. One of the major challenges to the efficacy of chemotherapy and targeted therapy is the development of cellular drug resistance. Thus, there is continued focus on development of novel agents to overcome the development of cellular drug resistance to current therapies and to enhance overall clinical efficacy.

Plants and other natural products have been an important resource for the discovery of novel anticancer therapeutic agents [3–8]. There is well-established evidence that our closest hominin relative, Neanderthals, used plant extracts for medicinal purposes [9]. In addition, plant extracts have been the basis for traditional Chinese medicine (TCM) for more than 2000 years as therapeutic treatments. PHY906, a 4-herb formulation, has been shown to reduce toxicities and enhance the antitumor activity of the topoisomerase I inhibitor irinotecan [10, 11]. Furthermore, our group identified a Huang Qin (HQ)-containing 5-herb formula (Huang Qin Ge Gen Tang (HQGGT)) that enhanced 5-fluorouracil (5-FU) antitumor activity through modulation of the E2F1/TS pathway [12]. However, it is unclear as to whether all of the herbal components contained within HQGGT are, in fact, required for 5-FU modulation.

In the present study, we identified HQ (*Scutellaria baicalensis radix*) as the main bioactive component of HQGGT. HQ is one of the major herbs listed in the Chinese Pharmacopoeia [13] containing over 300 compounds possessing antibacterial, antiviral, and antitumor activities [14]. We investigated the potential mechanisms of action and biological activities of HQ and its components in various CRC tumor models. Our results provide support for the role of HQ and the flavone baicalin as novel modulators of fluoropyrimidine chemotherapy in the treatment of drug-resistant CRC.

## Materials and methods

### Cell culture

Human colon cancer cells HT-29 (KRAS^wt^, BRAF^V600E^, TP53^R273H^), RKO (KRAS^wt^, BRAF^V600E^, TP53^wt^), and SW48 (KRAS^wt^, BRAF^wt^, TP53^wt^), and normal human colon epithelial CCD841 CoN cells were purchased from American Type Culture Collection (ATCC; Rockville, MD). The human colon cancer H630 cell line (KRAS^wt^, BRAF^wt^, TP53^mut^) was originally obtained from Dr. Adi Gazdar and maintained in our laboratory [15]. H630-R1 5-FU-resistant cells were previously established in our laboratory following chronic exposure to 5-FU [16]. The 5-FU IC_50_ value for H630-R1 cell line was 11-fold higher than that for the parent H630 cell line. 5-FU resistant RKO (RKO-R10) cells were established in our laboratory after chronic exposure to 5-FU and are grown in 10 µM 5-FU. The 5-FU IC_50_ value for RKO-R10 cell line was 50-fold higher than that for the parent RKO cell line. The parental HCT116 (KRAS^G13D^, BRAF^wt^, TP53^wt^), subclone HCT116 p53^(−/−)^ (p53 knockout), and HCT116 p21^(−/−)^ (p21 knockout) cell lines were kindly provided by Dr. B. Vogelstein (Johns Hopkins University). HCT116 PUMA^(−/−)^ (PUMA knockout) cell line was obtained from Dr. Lin Zhang (University of Pittsburgh). The mouse colon cancer cell line (MC38) was obtained from Dr. Michael Lotze (University of Pittsburgh). Cell lines were authenticated February 2016 by human STR profiling performed by the University of Pittsburgh Cytogenetics Facility and IDEXX BioResearch, respectively. Cells were tested monthly for mycoplasma by the e-Myco PLUS Mycoplasma PCR Detection Kit (Bulldog Bio, Portsmouth, NH, Cat#25237). Human cells were maintained in RPMI-1640 media (GIBCO; Grand Island, NY) and mouse cells (MC38) were maintained in DMEM media (GIBCO) supplemented with 10% fetal bovine serum (Gemini Bio-Products; Sacramento, CA) at 37 °C in a humidified incubator with 5% CO_2_.

### Solution preparation

Herb granules [(HQ, *Scutellaria baicalensis* Georgi Radix), Gegen (GG, *Pueraria lobata Ohwi* Radix), Shengma (SM, *Cimicifuga foetida L.* Rhizoma), Baishao (BS, *Paeonia lactiflora Pall* Radix) and Gancao (GC, *Glycyrrhiza uralensis Fisch* Radix)] were purchased from Sun Ten Pharmaceutical Co. (Taipei, Taiwan). The herb weight ratio for Huang Qin Ge Gen Tang formulation (HQGGT) was HQ:10 g; GG:15 g; SM:6 g; BS:10 g; GC:3 g as previously described [12]. Granules were weighed and resuspended in deionized water and incubated at 80 °C for 30 min. The supernatant was separated from any insoluble material by centrifugation (3000 × g, 20 min) and sterilized by passage through a 0.22 μm filter. For in vitro cell culture studies, 100 mg/mL HQ extract was prepared. For in vivo animal experiments, 300 mg/mL HQ (actual soluble weight is 200 mg/mL) and 20 mg/mL BI solution (DMSO-PBS solution) were used. Baicalin (BI), baicalein (BE), wogonin (WO), and wogonoside (WS) (Sigma; St. Louis, MO) were dissolved in DMSO (50 mM stock solution).

### HPLC analysis of HQ and its components

The chemical fingerprint of three different HQ batches (1: Lot#1070831, 2: Lot#T2081080, 3: Lot#3070715) and content of components BI, BE, WO, WS were measured by high performance liquid chromatography (HPLC) analysis as previously described [12]. Each batch was diluted to 50 µg/mL in acetonitrile/water (15:85 *v:v*), and 50 µL was injected into the HPLC–UV system. The LC system consisted of an Agilent (Palo Alto, CA, USA) 1100 autosampler, 1100 quaternary pump, and a 1260 variable wavelength detector. The method used a Waters Xterra column (5 µm, 250 × 4.6 mm) (Milford, MA USA), and a gradient mobile phase. Mobile phase solvent A consisted of acetonitrile, and mobile phase solvent B consisted of 0.1% phosphoric acid in water. The total run time was 70 min, and the 280 nm wavelength was used to monitor the eluent.

### Cell proliferation assay

Cells were plated in 96-well plates at a density of 800–3000 cells/well. On the following day, cells were incubated with extracts and compounds (HQ, GG, SM, BS, GC, BI, BE, WO, WS) for 72 h. Cell viability was quantified by the WST-1 assay (Roche; Indianapolis, IN). The IC_50_ value is defined as the concentration of drug required to inhibit cell growth by 50% when compared to untreated cells. All assays were performed in duplicate with at least 3–5 independent experiments.

### Combination index analysis

H630R1, RKO-R10, and MC38 cells were plated and treated with various concentrations of HQ, BI, 5-FU, 5’-deoxyfluorouridine (DFUR) (Sigma; St. Louis, MO). After 72 h, cell viability was measured by WST-1 assay. The Combination Index (CI) median effect analysis was performed to evaluate the effects of the combinations. A CI value less than, equal to, and more than 1 indicates synergy, additivity, or antagonism, respectively.

### Cell cycle analysis

The effect of HQ on cell cycle distribution was determined by flow cytometry analysis. Cells were incubated with HQ (1 mg/mL) for 48 h, followed by fixation, propidium iodide staining, and analyzed on a BD Accuri C6 flow cytometer.

### Reverse-phase protein array analysis

HCT116 cells were seeded into 6-well plates at a density of 2.0 × 10^5^ cells per well. On the following day, cells were treated with HQ (0.3 mg/mL) or BI (60 µM) for 48 h. Cells were harvested and protein lysates were prepared. Samples were sent to the Functional Proteomics RPPA Core Facility at the MD Anderson Cancer Center (Houston, TX) for analysis.

### Immunoblot analysis

Cells were treated with HQGGT, HQ, GG, SM, BS, GC, BI, BE, WO, and WS for 48 h and processed for immunoblot analysis as previously described [12]. The following primary antibodies (obtained from Cell Signaling Inc. unless noted) were used at the indicated dilutions: anti-TS, 1:2000 (#9045); anti-E2F1, 1:1000 (#3742); anti-GAPDH, 1:10,000 (#5174; #sc-47724, Santa Cruz); anti-p-NDRG1, 1:1,000 (#5482); anti-NDRG1, 1:1,000 (#9408); anti-p-P65, 1:5,000 (#3033); anti-P65, 1:5,000 (#8242); anti-PUMA, 1:1,000 (#4976); anti-p-RB, 1:1,000 (#9307); anti-RB, 1:1,000 (#9313); anti-CHK1, 1:1,000 (#2345); anti-HSP27, 1:1,000 (#2402); anti-CyclinB1, 1:1,000 (#12231); anti-STAT3, 1:5,000 (#9139); anti-MCL1, 1:1,000 (#5453); anti-CDK4, 1:1,000 (#12790); anti-CDK6, 1:1,000 (#3136); anti-CyclinD1, 1:1,000 (#2978); anti-DHFR, 1:1,000 (#610696, BD Biosciences); anti-TK1, 1:1,000 (#40688, Novus Biologicals). Proteins were detected using SuperSignal West Pico substrate (Pierce; Rockford, IL). Quantitation of signal intensities was performed by densitometry on a HP scanner using NIH IMAGEJ software.

### NF-κB activity analysis

RKO cells were transfected with pGL4.32 plasmid (Promega; Cat. #E849A) containing luciferase under the control of a NF-κB response element. Stable expression was established following 2 weeks of hygromycin exposure. RKO cells were treated with HQ or its components BI, BE and WO for 48 h. Luciferase activity was determined in cell lysates using the Promega Luciferase Assay system (Cat# E1910) and normalized by the protein concentration of the cell lysate.

### Real time quantitative reverse transcription PCR (qRT-PCR)

Cells were treated with HQ (0.5 mg/mL) for 48 h. Total RNA was extracted in Trizol (Invitrogen; Carlsbad, CA). qRT-PCR analysis was performed as previously described [12]. RNA levels of RB, TS, E2F1, and 18S were assessed using the TaqMan Gene Expression real-time PCR assays (Applied Biosystems assay IDs: Hs01078066_m1; Hs00426586_m1; Hs00153451_m1; Hs03928990_g1). Results were expressed as the threshold cycle (Ct). The relative quantification of target transcripts was determined by the comparative Ct method (ΔΔCt) according to the manufacturer’s protocol. The 2^−ΔΔCt^ method was used to analyze the relative changes in gene expression. Control PCR experiments in the absence of reverse transcription were performed to confirm that the total RNA was not contaminated with genomic DNA.

### In vivo mouse xenograft model

The animal study protocol was approved by the Institutional Animal Care and Use Committee (IACUC) of the University of Pittsburgh and in accordance with the National Institutes of Health Guide for the Care and Use of Laboratory Animals. MC38 cells (2 × 10^6^ cells per mouse) were inoculated subcutaneously into the right flank of C57BL/6 mice. Once tumors reached a size of approximately 100 mm^3^, mice were randomized into 4 groups (7 mice per group): (A) vehicle control (200 µL sterile water), p.o. qdx5/week; (B) HQ, 4 g/kg/day body weight, (2 g/kg, p.o. twice a day, qdx5/week); (C) 5-FU, 50 mg/kg, i.p. weekly for 6 weeks, and (D) HQ in combination with 5-FU. To evaluate the antitumor effect of HQ in combination with capecitabine (CAP, Sigma, St. Louis, MO), tumor-bearing mice were randomized into 4 groups (7 mice per group): (A) vehicle control (water), p.o. qdx5/week; (B) HQ, 2 g/kg, p.o. daily; (C) CAP, 200 mg/kg body weight, p.o. 7 days on/7 days off for 6 weeks, and (D) HQ in combination with CAP. To evaluate the antitumor effect of BI in combination with 5-FU, MC38-bearing mice were randomized into 4 groups (5 mice per group): (A) vehicle control (DMSO/PBS), IP qdx3/week; (B) BI, 100 mg/kg body weight, IP qdx3/week; (C) 5-FU, 50 mg/kg body weight, IP weekly, and (D) BI in combination with 5-FU. Tumor volume (mm^3^) was calculated using the formula: 1/2(L × W^2^) where L is the longest and W is the shortest axis.

### Statistical analysis

Data are presented as mean ± S.D. unless otherwise indicated. The Student’s t-test (two-tailed) was used to determine statistical significance between two groups. For comparisons between groups of more than two unpaired values, one-way analysis of variance (ANOVA) was used. Tumor response to treatment was compared using two-way ANOVA, post-test Bonferroni. Analysis was done with Prism version 6 (GraphPad Software, Inc.). Values of *p* < 0.05 were considered statistically significant.

## Results

### HQ is the main bio-active component of HQGGT formula

We had previously demonstrated that a 5-herb formula, HQGGT, enhanced the in vivo antitumor activity of 5-FU through inhibition of the E2F1/thymidylate synthase (TS) protein [12]. To determine the relative contribution of each herb to the cytotoxic effects of the HQGGT formula, IC_50_ values were determined in several human CRC cell lines after sequentially removing one herb from the formula. As seen in Table 1, removal of HQ from the formula had the most significant effect on reducing overall cytotoxicity. When each herb was tested individually, HQ displayed the greatest inhibitory effects on CRC cell growth.

**Table 1:** Cytotoxicity of HQ and other individual herbs of HQGGT formulation

Formulation					IC_50_ (mg/ml)			
HQ	GG	SM	BS	GC	HT-29	RKO	HCT116	HCT116 p53(-/-)
+	+	+	+	+	1.51 ± 0.22	0.96 ± 0.19	0.78 ± 0.13	0.46 ± 0.07
**-**	** + **	** + **	** + **	** + **	**4.20 ± 0.06**	**1.03 ± 0.37**	**1.80 ± 0.19**	**1.16 ± 0.24**
+	-	+	+	+	1.04 ± 0.08	0.36 ± 0.03	0.31 ± 0.04	0.24 ± 0.01
+	+	-	+	+	2.02 ± 0.11	0.57 ± 0.01	0.52 ± 0.04	0.41 ± 0.04
+	+	+	-	+	2.09 ± 0.06	0.59 ± 0.19	0.47 ± 0.05	0.42 ± 0.05
+	+	+	+	-	2.21 ± 0.10	0.74 ± 0.17	0.63 ± 0.09	0.52 ± 0.03
Individual Herbs								
** + **	**-**	**-**	**-**	**-**	**0.50 ± 0.06**	**0.10 ± 0.01**	**0.09 ± 0.01**	**0.05 ± 0.01**
-	+	-	-	-	5.06 ± 1.28	1.17 ± 0.22	2.97 ± 1.65	2.62 ± 1.77
-	-	+	-	-	2.31 ± 0.23	0.71 ± 0.14	1.14 ± 0.32	0.82 ± 0.14
-	-	-	+	-	3.53 ± 0.08	1.16 ± 0.02	1.08 ± 0.30	1.12 ± 0.21
-	-	-	-	+	3.00 ± 0.87	1.57 ± 0.06	2.06 ± 0.10	2.00 ± 0.06

All IC_50_ values represent the mean ± SD from 3–5 independent experiments

**Fig. 1:**
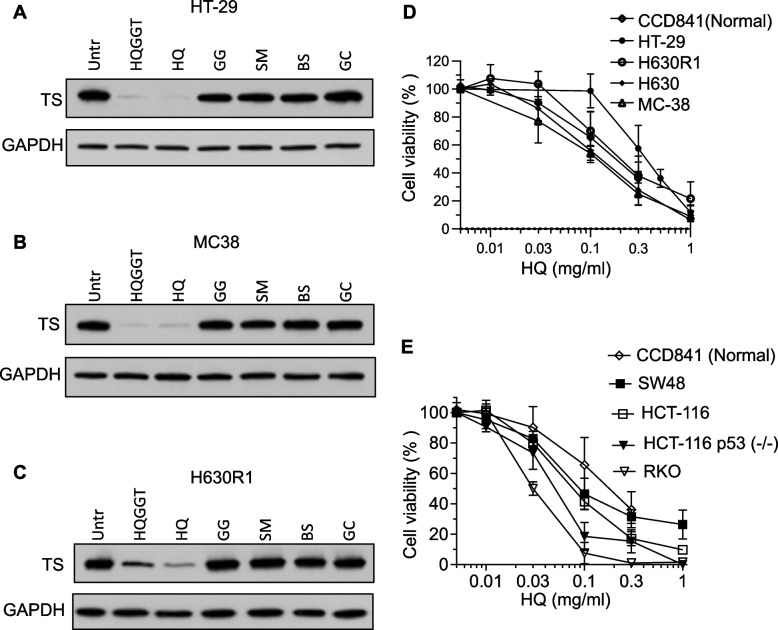
Effect of HQ on TS expression and cell proliferation. HT-29 (**A**), MC38 (**B**) and H630-R1 (**C**) cells were treated with HQGGT (3 mg/ml), HQ (0.5 mg/ml), GG (1.2 mg/ml), SM (0.5 mg/ml), BS (0.5 mg/ml), GC (0.2 mg/ml) for 48 h and processed for immunoblot analysis. **D**, **E** Cells were treated with HQ for 72 h. Cell viability was measured by WST-1 assay from 3–5 separate experiments

To identify the specific herb in HQGGT that was directly responsible for inhibiting expression of TS, CRC cells were incubated with either the parent formula HQGGT or equivalent single herb concentration as in the formula. As seen in Fig. 1A-C, HQ was the only herb that suppressed TS protein expression.

### HQ exhibited in vitro antiproliferative activity against CRC cells

The effect of HQ on proliferation of CRC cells with different genetic backgrounds was analyzed. Cells were incubated with increasing concentrations of HQ (0.01–1.0 mg/mL) for 72 h. HQ treatment resulted in concentration-dependent cell growth inhibition (Fig. 1D, E, Additional file 2: Table S1). Of note, chemo-resistant cell lines (H630R1; RKOR10) were equally sensitive to HQ mediated growth inhibition. Similarly, there was little to no difference in IC_50_ values between HCT116, HCT116 p53^−/−^, HCT116 p21^−/−^ and HCT116 PUMA^−/−^ cells. These results suggest that HQ displays antiproliferative activity against drug resistant CRC cells as well as against cells with different genetic backgrounds. Growth of normal colon epithelial cells (CCD841) was also inhibited by HQ treatment suggesting a universal antiproliferative effect on cultured cell lines (Fig. 1D, E, Additional file 2: Table S1).

### Batch to batch variation

One of the main issues with herbal and plant extracts is variability in the manufacturing process resulting in different biological effects. To document batch-to-batch consistency for the commercial source, we evaluated three different batches of HQ, obtained over a three-year period, for their ability to inhibit CRC cell growth. Treatment of CRC cells with each HQ batch resulted in nearly identical IC_50_ values (Additional file 2: Table S2). In addition, the major flavones present in HQ (baicalin, baicalein, wogonoside, and wogonin) were consistently detected in each batch with a coefficient of variation of less than 3% (Additional file 2: Table S3). These results provide support for the reliability of the herb with consistent biological effects, the reproducibility of the extraction process, and the stability of the major individual herbal components.

### HQ promoted CRC cell apoptosis

To further investigate the biological effects of HQ on CRC cells, cell cycle distribution was determined by flow cytometry analysis following HQ incubation. Treatment of HT-29 cells with HQ (1 mg/mL) for 48 h significantly increased the number of cells in the sub-G0 phase compared with untreated cells (Additional file 1: Fig. S1A-B). Similarly, HQ treatment resulted in a significantly higher proportion of HCT116, RKO, and 5-FU-resistant H630R1 cells in the sub-G0 phase (Additional file 1: Fig. S1C). Since the sub-G0 phase reflects the population of cells that are undergoing apoptosis, these findings confirm that HQ treatment is associated with induction of apoptosis in CRC cells as previously reported [17, 18].

### Effect of HQ on NF-κB and CDK-RB signaling pathways

**Fig. 2:**
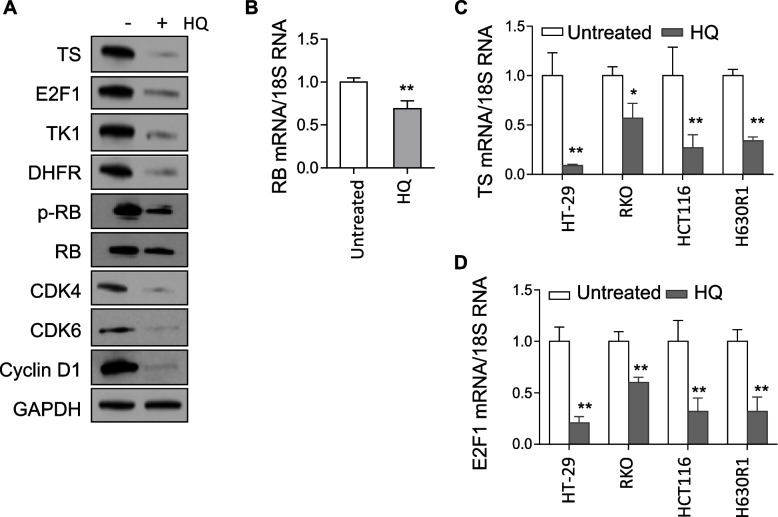
Effect of HQ on Rb/E2F1/TS signaling pathway. **A** HT29 cells were treated with 0.5 mg/ml HQ for 48 h and processed for immunoblot and qRT-PCR analysis. The mRNA levels of RB (**B**), TS (**C**), and E2F1 (**D**) were decreased following 48 h HQ treatment. Values represent the mean ± SD from 3 independent experiments. *, *p* < 0.05, **, *p* < 0.01, versus untreated control

To gain greater insight into the potential signaling pathways affected by HQ treatment, we employed the reverse-phase protein microarray (RPPA) assay as an initial screen to investigate both total protein and phosphoprotein expression. We found alterations in expression of several cellular proteins in HT29 cells after HQ treatment (Additional file 2: Table S4). These protein changes were confirmed by immunoblot analysis (Additional file 1: Fig. S2A, B). Of note, we observed a 51% increase in expression of p-NF-κB p65 protein. To validate this observation, we measured NF-κB activity by utilizing a luciferase reporter assay under the control of a NF-κB response element. Treatment with HQGGT and HQ, respectively, stimulated NF-κB activity, in a concentration-dependent manner (Additional file 1: Fig. S2C). HQ incubation also resulted in reduction in protein expression. As shown in Figs. 1 and 2A, treatment with HQ significantly decreased the expression of TS protein in CRC cells. Transcription of TS and other S phase proteins such as TK1 and DHFR has been shown to be regulated, at least in part, by E2F1 transcription factor [19–21]. Following HQ treatment, TS and E2F1 protein levels decreased significantly along with TK1 and DHFR (Fig. 2A). RB is a tumor suppressor protein that binds and controls the function of E2F1. HQ treatment reduced p-RB and RB expression (Fig. 2A). These reductions in expression of RB and TS were found to be both time- and concentration-dependent (Additional file 1: Fig. S3A-B). Of note, these protein changes were similar in response to treatment with the different HQ batches. (Additional file 1: Fig. S3C). To further identify the key signaling pathways mediated by HQ, we analyzed the expression of upstream cellular proteins known to regulate RB (CDKs, cyclins) and found that CDK4, CDK6, and cyclin D1 protein levels were significantly decreased after HQ treatment (Fig. 2A). In addition to alterations in protein expression, HQ treatment resulted in a significant decrease in the mRNA levels of RB, TS, and E2F1 (Fig. 2B-D) suggesting the possibility of a transcriptional control mechanism. Together, these findings suggest that the growth inhibitory activity of HQ is likely due to inhibition of the CDK-RB pathway.

### HQ enhanced colon cancer cell sensitivity to 5-FU

**Fig. 3:**
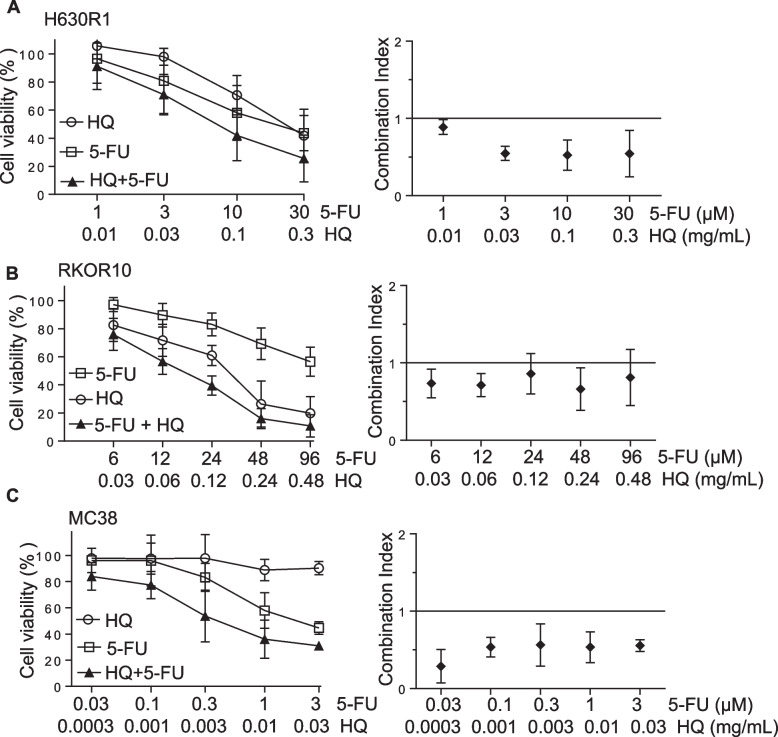
Effect of HQ in combination with 5-FU on CRC cell proliferation. H630R1 (**A**), RKOR10 (**B**) and MC38 (**C**) cells were treated with various concentrations of HQ and 5-FU for 72 h. Cell viability was measured by WST-1 assay (left panel). The Combination-Index (CI) was calculated with CI < 1 indicating synergism (right panel) from at least 3 individual experiments performed in duplicate

Previously, we had shown that the herbal formula HQGGT in combination with 5-FU had a synergistic anti-proliferative effect on both human and mouse CRC cells [12]. We evaluated whether HQ alone was able to enhance 5-FU cytotoxicity. Human 5-FU-resistant colorectal cancer cells H630-R1 and RKO-R10 were treated with various concentrations of HQ alone or in combination with 5-FU. Following 72-h treatment, cell viability was assessed by WST-1 assay. As seen in Fig. 3A and B, the combination resulted in enhanced cell growth inhibition. The combination index (CI) was < 1 indicating a synergistic effect between HQ and 5-FU. In addition, we observed that the combination of HQ and 5-FU was synergistic in mouse MC38 colorectal cancer cells (Fig. 3C).

Upon intracellular uptake, 5-FU is metabolized to the fluoropyrimidine metabolite FdUMP, which, in turn, binds to TS protein eventually leading to an increase in TS protein synthesis resulting from abrogation of the normal translational autoregulatory mechanism [16]. Our group has previously shown that the acute induction of TS protein induction leads to the development of acute 5-FU resistance. We evaluated the effect of HQ on this resistance mechanism. Treatment of HT29 cells with 5-FU induced TS protein expression, while HQ treatment significantly decreased TS protein (Additional file 1: Fig. S3D). 5-FU treatment also induced a slower migrating band at ~38 kDa. This band corresponds to the presence of the covalent inhibitory ternary complex (ITC), which is made up of TS protein, the reduced folate 5,10-methylenetetrahydrofolate and FdUMP [22]. The combined treatment of 5-FU with HQ resulted in reduced expression of both TS protein and ITC. These findings suggest that the synergistic anti-proliferative effect of the combination on CRC cells may be mediated through suppression of the 5-FU-mediated induction of TS.

### HQ enhanced the anti-tumor effect of 5-FU and capecitabine in vivo

**Fig. 4:**
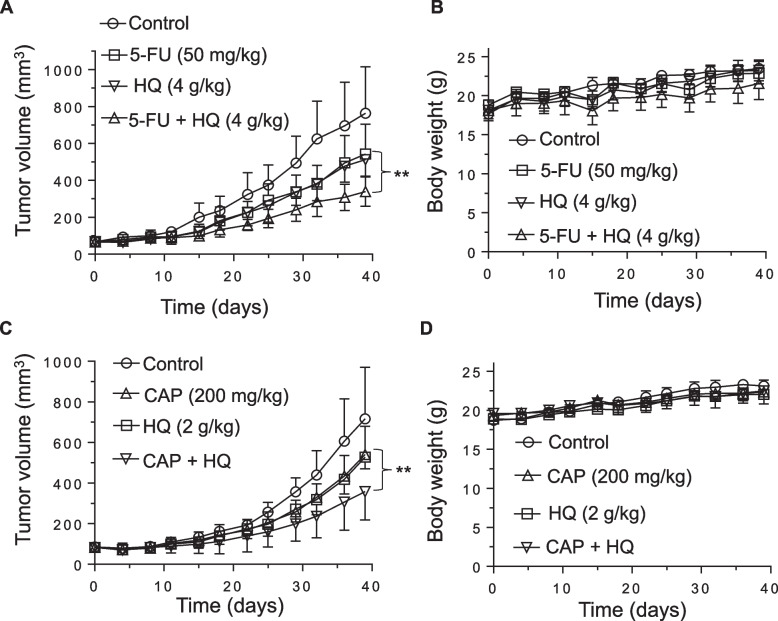
Effect of HQ in combination with 5-FU or capecitabine on tumor growth. HQ was orally administered twice a day, 5 times a week, and 5-FU was i.p. administered once a week for 6 weeks to MC38 tumor-bearing C57BL/6 mice. Tumor volume (**A**) and body weight (**B**) were determined twice a week. **C**, **D** HQ was administered daily, and capecitabine (CAP) was orally administered 7 days on/7 days off for 6 weeks to MC38-bearing C57BL/6 mice. Tumor volume (**C**) and body weight (**D**) were determined twice a week. Measurements represent the mean ± SD (7 mice per group). **, *p* < 0.01

Based on the promising growth inhibitory effects of HQ in combination with 5-FU in vitro, we next investigated the efficacy of this combination in vivo. Mice bearing MC38 allografts were randomized into 4 groups (7 mice per group): vehicle; HQ alone; 5-FU alone; and HQ plus 5-FU. As shown in Fig. 4A, daily administration of HQ or weekly 5-FU administration resulted in a slight reduction of tumor growth. The combination of HQ and 5-FU suppressed tumor growth to a significantly greater extent than HQ or 5-FU alone (*p* = 0.0021, *p* = 0.0002, respectively). No body weight loss was observed (Fig. 4B) and no morphological changes were observed in liver and intestine following HQ treatment (Additional file 1: Fig. S4). Markers of proliferation and apoptosis, Ki-67 and TUNEL, remained unchanged in these normal tissues following HQ treatment (Additional file 1: Fig. S4). The oral pro-drug formulation of 5-FU, capecitabine (CAP), is widely used in clinical practice in the U.S. and throughout the world. Prior to combining HQ with CAP in vivo, we evaluated this combination in vitro. As most cancer cells lack the carboxylesterase and cytidine deaminase necessary to convert CAP to 5’-deoxy’5-fluorouridine (DFUR), MC38 cells were treated with the combination of HQ and DFUR. We observed significant in vitro synergy with this combination (Additional file 1: Fig. S5). Given this synergy, mice bearing MC38 allografts were randomized into 4 groups (7 mice per group): vehicle; HQ (2 g/kg); CAP (200 mg/kg); and HQ plus CAP. As shown in Fig. 4C, a slight reduction of tumor growth was observed following. Compared with HQ or CAP alone, the combination of HQ and CAP significantly inhibited tumor growth (*p* = 0.0003, *p* < 0.0001, respectively). No body weight loss was observed with these treatments (Fig. 4D). These findings suggest that HQ can enhance the antitumor activity of both 5-FU and CAP in our in vivo mouse models.

### Active components of HQ

Given the complexity and difficulty of using plant extracts as therapeutic agents, a reductionist approach was taken in an attempt to identify the active component of HQ. The main active components of HQ are thought to be flavones along with their glucuronidated metabolites. Using HPLC/UV, we detected the four main flavones (baicalin (BI), baicalein (BE), wogonin (WO), wogonoside (WS)) and found that baicalin routinely comprised ~ 11% of HQ, which was considerably higher than other flavones (0.19–2%) (Additional file 2: Table S3). The effect of single flavones on cell proliferation was analyzed. IC_50_ values of BI, BE, and WO against human HT-29 cancer cells was 92, 68, and 85 µM, respectively (Additional file 2: Table S5). No growth inhibitory effects were observed following incubation with 300 µM WS (the highest soluble concentration).

**Fig. 5:**
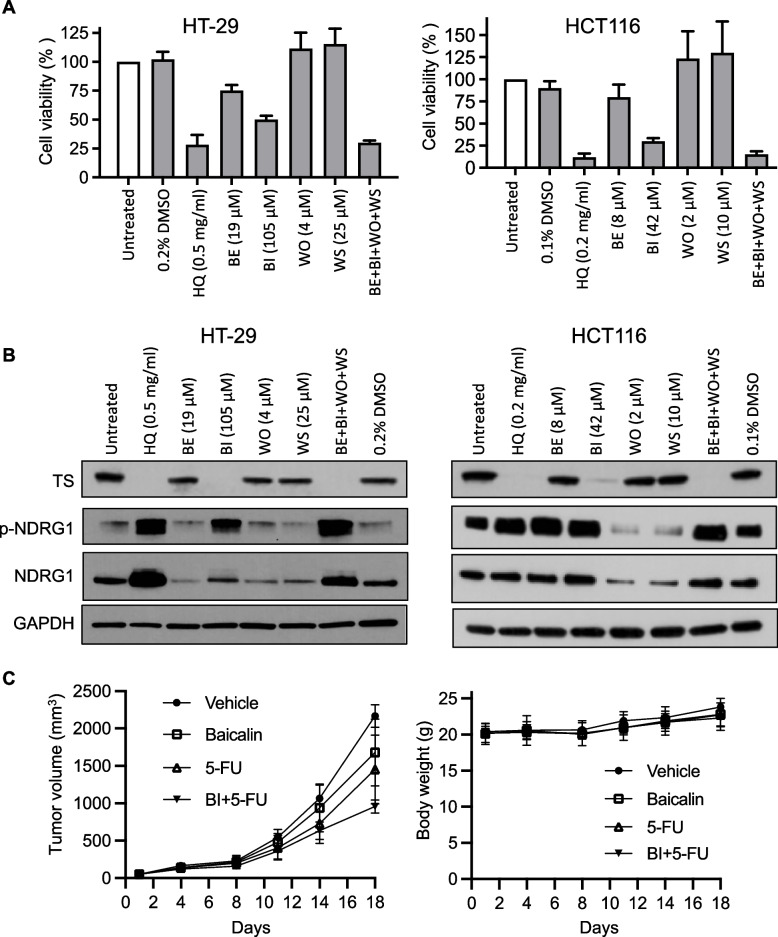
Effect of HQ and its main flavones on CRC growth and protein expression. **A** HT-29 and HCT116 cells were treated with HQ, baicalin (BI), baicalein (BE), wogonin (WO), wogonoside (WS) for 72 h. Cell viability was measured by WST-1 assay. **B** HT-29 and HCT116 cells were treated with HQ and the flavones for 48 h and processed for immunoblot analysis. **C** BI was IP administered (100 mg/kg) three times a week, and 5-FU was i.p. administered (50 mg/kg) once a week for 3 weeks to MC38 tumor-bearing C57BL/6 mice. Tumor volume and body weight were determined twice a week. Measurements represent the mean ± SD (5 mice per group)

To identify which flavones contribute significantly towards HQ in vitro cytotoxicity, we compared the effect of HQ alone to that of the individual flavones at the concentration present in HQ. As seen in Fig. 5A, baicalin had the most pronounced effect on cell growth. Combining all 4 flavones resulted in cell growth inhibition equivalent to HQ extract. These four flavones were tested for their ability to alter protein expression. We identified baicalin as an active component in HQ that was directly responsible for inhibition of TS protein and increasing p-NDRG1 expression (Fig. 5B). In addition, we evaluated the effect of the individual flavones on NF-κB activity by utilizing a luciferase reporter assay. The results showed that both baicalin and baicalein stimulated NF-κB activity whereas wogonin had no effect (Additional file 1: Fig. S2D). We next investigated the ability of baicalin to restore 5-FU sensitivity to RKOR10 cells. Baicalin combined with 5-FU demonstrated synergistic activity against 5-FU-resistant RKO-R10 cells (Additional file 1: Fig. S6).

To further evaluate the mechanistic contribution of BI, we performed RPPA analysis comparing HQ with BI treatment. The observed changes in cellular protein expression were similar between HQ and baicalin treatment. Only two proteins were differentially altered, AXL and TFRC (Additional file 1: Fig. S7A-B). Together, these findings suggest that baicalin is one of the main components of HQ responsible for the in vitro biological activity against CRC cells.

Based on the promising growth inhibitory effects of BI in combination with 5-FU in vitro, we investigated the efficacy of this combination in vivo. MC38-bearing mice were randomized into 4 groups: vehicle; oral BI alone at 0.6 g/kg daily (equivalent to 4 g/kg HQ); 5-FU alone 50 mg/kg weekly; and BI plus 5-FU. Unfortunately, oral administration of BI had no effect on tumor growth and did not enhance the activity of 5-FU (data not shown). This unexpected finding may be related to the limited oral bioavailability of BI itself [23]. Given this result, we repeated these experiments with IP BI administration (3 times a week; 100 mg/kg). Administration of BI by itself had minimal effect on tumor growth (~ 20%; *p* = 0.049 vs. vehicle) while 5-FU alone had a modest 30% suppressive effect on growth (*p* = 0.011 vs. vehicle) (Fig. 5C). However, the combination of BI and 5-FU significantly inhibited tumor growth by ~60% (*p* = 3E-07 vs. vehicle).

## Discussion

*Scutellaria baicalensis radix* (Huang Qin; HQ) is one of the most widely used herbs in Asia, and it is used either alone or in multi-herb formulations to treat a wide range of medical indications. We previously demonstrated that an HQ-containing herbal formulation HQGGT enhanced the antitumor activity of 5-fluorouracil, a standard CRC chemotherapy agent [12]. Other HQ-based formulas, such as Huang Qin Tang (PHY906) have been shown to reduce the gastrointestinal side effects associated with irinotecan-based chemotherapy [10, 11] and to enhance the antitumor effect of capecitabine and decrease the toxicities associated with chemotherapy and radiation therapy [24–26]. Herein, we investigated the active component of HQGGT and determined that HQ was responsible for the main biological activities with the other 4 herbs contributing little to the formula activity. HQ treatment inhibited expression of CDK4/RB/E2F1 proteins leading to downregulation of key S-phase proteins such as TS. This reduction led to enhanced antiproliferative activity in combination with 5-FU and its oral prodrug capecitabine in both in vitro and in vivo model systems.

Although herbal medicines have been widely used throughout the world, to date, no herbal extracts have been approved by the U.S. FDA for oral administration. In general, the clinical studies to investigate herbal medicines have been poorly designed with ill-defined methodologies as well as serious quality control issues regarding the extract source and characterization. In the present study, as well as in our previously published work, we obtained herb extracts from a well-established source (Sun Ten Pharmaceutical Co.) over several years and found that the main components and the associated biological activities were consistent between different herb batches. This finding provides confidence with respect to the biological reproducibility as we move forward toward human clinical studies. However, we sought to identify the active component(s) in the HQ extract responsible for the synergy with fluoropyrimidine therapy. Among the most highly represented flavones within the extract, we discovered that baicalin, a flavone glycoside, had similar in vitro biological activity as the HQ extract itself. An RPPA analysis demonstrated a comparable pattern of protein alterations following incubation with either HQ or baicalin. Of note, HQ was broadly cytotoxic against all CRC cell lines and normal colon cells. All four of the main HQ flavones, when evaluated individually, had modest cytotoxicity at high micromolar concentrations. However, we observed no toxicity when HQ was administered to mice. This is likely due to first pass metabolism of HQ that alters the extract components and concentrations leading to differential exposures between mouse tissues and cultured cells.

Baicalin and its aglycone baicalein have been extensively studied for their anti-apoptotic, anti-inflammatory, anti-steatosis, and anti-proliferative activities [17, 18, 27, 28]. Oral administration of baicalein has been found to be safe and well tolerated by healthy volunteers [29, 30]. The results presented herein suggest that inhibition of cell growth and proliferation in CRC cells by baicalin is mediated through multiple proliferative pathways including the CDK/RB signaling pathway. However, we were unable to demonstrate a direct interaction between baicalin and these cellular proteins (Additional file 1: Fig. S7). Zhang et. al. demonstrated that baicalein inhibited the DNA mismatch repair pathway by binding to MSH2 and MSH6 proteins [28]. In our study, we were unable to identify any significant interaction between baicalin and these DNA repair proteins (Additional file 1: Fig. S7). Furthermore, it has been suggested that the anti-inflammatory activity of these flavones is due to suppression of NF-κB [31, 32]. In our studies, we showed that the HQGGT formula, the HQ extract, and the two flavones baicalin, and baicalein actually increased phosphorylation of NF-κB and its transcriptional activity. While our differential findings may be related to our cell model systems, others have shown similar activation of NF-κB with these flavones [33, 34]. Samuel et. al. reported that the activation of NF-κB from various chemotherapeutic agents depended upon the cellular genetic makeup [35]. With the reported anti-inflammatory potential of HQ and the flavones, we utilized MC38-bearing immune-competent mice to demonstrate enhancement of 5-FU activity by HQ and BI. Future studies will examine the role of BI and HQ on immune cell activity as it relates to tumor growth.

Several investigators have demonstrated antitumor activity with both baicalin and baicalein [17, 18, 31, 36–38]. We observed modest tumor growth inhibition with baicalin itself but achieved significant suppression of tumor growth with the combination of baicalin and 5-FU. Oral administration of baicalin was unable to enhance the antitumor activity of 5-FU, unlike oral HQ. Baicalin, itself, is not readily absorbed by the intestine following oral administration. However, it is readily absorbed once metabolized to baicalein by b-glucuronidase in the gut bacteria [23, 39]. The majority of baicalein is then converted back to baicalin for systemic circulation. Various studies have demonstrated that baicalin absorption from herb extract such as HQ depends on the other components within the mixture [40–43]. The interplay between the hundreds of compounds within a plant extract can have additive, synergistic, or even antagonistic effects on the observed biological activity [44]. Of note, while HQ as a single agent enhanced the antitumor activity of 5-FU, the 5-herb formula HQGGT had a more substantial effect on tumor growth [12]. Thus, the multi-component mixture may have improved baicalin absorption and/or prevented its degradation or worked in concert with baicalin to enhance the antitumor activity.

In conclusion, we have identified HQ and its component baicalin that are responsible for its enhancement of fluoropyrimidine antitumor activity, mediated through suppression of the various signaling pathways such as CDK/RB. Future studies will examine the influence of other HQ components on baicalin uptake and metabolism as it pertains to 5-FU modulation. Our results provide support for the role of HQ and baicalin as novel modulators of fluoropyrimidine chemotherapy in the treatment of drug-resistant CRC.

## Supplementary Information

**Additional file 1: Fig. S1.** Effect of HQ on cell cycle distribution.HT-29 cells were treated with HQ for 48 hr, followed by fixation, PI staining, and cell cycle analysis by flow cytometry.The percentage of HT-29 cells in sub G0, G0/G1, S, G2/M phases.The percentage of HCT116, RKO and H630R1 cells in sub G0 phase. Values represent the mean ± S.D. from three independent experiments. **, *p* < 0.01, versus untreated. **Fig. S2.** Effect of HQ on signaling pathways.HT29 cells were treated with 0.5 mg/ml HQ for 48 hr and processed for immunoblot analysis.RKO cells stably expressing NF-kB-regulated luciferase were treated with HQ, HQGGT and individual flavonesfor 48 hr. Luciferase activity was determined and normalized to total soluble protein. **Fig. S3.** Time and dose dependent effects of HQ on RB and TS.HT-29 cells were treated with HQfollowed by processing for immunoblot analysis.HT-29 cells were treated with various concentrations of HQ for 48 hr and processed for immunoblot analysis.HT-29 cells were treated with three different HQ batches for 48 hr, respectively, and processed for immunoblot analysis.HT-29 cells were treated with HQ for 24 hr, then 5-FU was added for another 24 hr and processed for immunoblot analysis. Batch 1: lot#1070831, Batch 2: lot#T2081080, Batch 3: lot#3070715. **Fig. S4.** Effect of HQ on normal tissues*.* HQwas orally administered QD × 5 for 6 weeks to MC38-bearing C57BL/6 mice. Formalin-fixed sections of the liver and middle jejunum were stained with hematoxylin and eosin, Ki-67, and TUNEL. Scale bars are 100 μm. **Fig. S5.** Effect of HQ in combination with DFUR on CRC cell proliferation. MC38 cells were treated with HQ and DFUR for 72 hr. Cell viability was measured by WST-1 assay. The Combination-Indexwas calculated with CI < 1 indicating synergism. **Fig. S6.** Effect of BI in combination with 5-FU on CRC cell proliferation. RKOR10 cells were treated with various concentrations of BI and 5-FU for 72 hr. Cell viability was measured by WST-1 assay. The Combination-Indexwas calculated with CI < 1 indicating synergism between BI and 5-FU. **Fig. S7.** Alterations in protein expression by HQ and BI. HCT116 cells were treated with HQor BIfor 48 hr. Cells were processed for RPPA analysis.Similar protein changes after HQ and BI treatment.Protein differences between HQ and BI treatment. Percent changes represent the mean from duplicate sample. Cutoff values for alterations were set at 25% for either treatment. **Fig. S8.** Effect of baicalin on protein stability. HT29and HCT116cells were incubated with 500 µM baicalin for 4 hr at 37ºC. Cells were processed for CETSA assay as described.

**Additional file 2: Table S1.** HQ IC_50_ values in CRC cells. **Table S2.** IC_50_ values of HQ against colon cancer cell lines. **Table S3.** Flavone concentration in three different HQ batches. **Table S4.** Proteins altered following HQ treatment. **Table S5.** Flavone IC_50_ values in HT-29 cells.

## Data Availability

Data sharing is not applicable to this article as no datasets were generated or analyzed during the current study.

## References

[1] Siegel RL, Miller KD, Fuchs HE, Jemal A (2022). Cancer statistics, 2022. CA Cancer J Clin.

[2] Venook AP, Niedzwiecki D, Lenz HJ, Innocenti F, Fruth B, Meyerhardt JA (2017). Effect of first-line chemotherapy combined with cetuximab or bevacizumab on overall survival in patients with KRAS wild-type advanced or metastatic colorectal cancer: a randomized clinical trial. JAMA.

[3] Cassileth B, Yeung KS, Gubili J (2008). Herbs and other botanicals in cancer patient care. Curr Treat Options Oncol.

[4] Gordaliza M (2007). Natural products as leads to anticancer drugs. Clin Transl Oncol.

[5] Harvey AL (2008). Natural products in drug discovery. Drug Discov Today.

[6] Khanfar MA, Alqtaishat S (2021). Discovery of potent natural-product-derived SIRT2 inhibitors using structure-based exploration of SIRT2 pharmacophoric space coupled with QSAR analyses. Anticancer Agents Med Chem.

[7] Lasso P, Gomez-Cadena A, Uruena C, Donda A, Martinez-Usatorre A, Romero P (2020). An immunomodulatory gallotanin-rich fraction from Caesalpinia spinosa enhances the therapeutic effect of anti-PD-L1 in melanoma. Front Immunol.

[8] Mohinudeen I, Kanumuri R, Soujanya KN, Shaanker RU, Rayala SK, Srivastava S (2021). Sustainable production of camptothecin from an Alternaria sp. isolated from Nothapodytes nimmoniana. Sci Rep.

[9] Weyrich LS, Duchene S, Soubrier J, Arriola L, Llamas B, Breen J (2017). Neanderthal behaviour, diet, and disease inferred from ancient DNA in dental calculus. Nature.

[10] Kummar S, Copur MS, Rose M, Wadler S, Stephenson J, O’Rourke M (2011). A phase I study of the chinese herbal medicine PHY906 as a modulator of irinotecan-based chemotherapy in patients with advanced colorectal cancer. Clin Colorectal Cancer.

[11] Lam W, Jiang Z, Guan F, Hu R, Liu SH, Chu E (2014). The number of intestinal bacteria is not critical for the enhancement of antitumor activity and reduction of intestinal toxicity of irinotecan by the Chinese herbal medicine PHY906 (KD018). BMC Complement Altern Med.

[12] Liu H, Liu H, Zhou Z, Parise RA, Chu E, Schmitz JC (2018). Herbal formula Huang Qin Ge Gen Tang enhances 5-fluorouracil antitumor activity through modulation of the E2F1/TS pathway. Cell Commun Signal.

[13] Zhao Q, Chen XY, Martin C (2016). Scutellaria baicalensis, the golden herb from the garden of Chinese medicinal plants. Sci Bull (Beijing).

[14] Shang X, He X, He X, Li M, Zhang R, Fan P (2010). The genus Scutellaria an ethnopharmacological and phytochemical review. J Ethnopharmacol.

[15] Park JG, Oie HK, Sugarbaker PH, Henslee JG, Chen TR, Johnson BE (1987). Characteristics of cell lines established from human colorectal carcinoma. Cancer Res.

[16] Copur S, Aiba K, Drake JC, Allegra CJ, Chu E (1995). Thymidylate synthase gene amplification in human colon cancer cell lines resistant to 5-fluorouracil. Biochem Pharmacol.

[17] Kim DH, Hossain MA, Kang YJ, Jang JY, Lee YJ, Im E (2013). Baicalein, an active component of Scutellaria baicalensis Georgi, induces apoptosis in human colon cancer cells and prevents AOM/DSS-induced colon cancer in mice. Int J Oncol.

[18] Tao Y, Zhan S, Wang Y, Zhou G, Liang H, Chen X (2018). Baicalin, the major component of traditional Chinese medicine Scutellaria baicalensis induces colon cancer cell apoptosis through inhibition of oncomiRNAs. Sci Rep.

[19] Kasahara M, Takahashi Y, Nagata T, Asai S, Eguchi T, Ishii Y (2000). Thymidylate synthase expression correlates closely with E2F1 expression in colon cancer. Clin Cancer Res.

[20] Koushyar S, Economides G, Zaat S, Jiang W, Bevan CL, Dart DA (2017). The prohibitin-repressive interaction with E2F1 is rapidly inhibited by androgen signalling in prostate cancer cells. Oncogenesis.

[21] Sowers R, Toguchida J, Qin J, Meyers PA, Healey JH, Huvos A (2003). mRNA expression levels of E2F transcription factors correlate with dihydrofolate reductase, reduced folate carrier, and thymidylate synthase mRNA expression in osteosarcoma. Mol Cancer Ther.

[22] Carreras CW, Santi DV (1995). The catalytic mechanism and structure of thymidylate synthase. Annu Rev Biochem.

[23] Akao T, Kawabata K, Yanagisawa E, Ishihara K, Mizuhara Y, Wakui Y (2000). Baicalin, the predominant flavone glucuronide of scutellariae radix, is absorbed from the rat gastrointestinal tract as the aglycone and restored to its original form. J Pharm Pharmacol.

[24] Lam W, Bussom S, Guan F, Jiang Z, Zhang W, Gullen EA (2010). The four-herb Chinese medicine PHY906 reduces chemotherapy-induced gastrointestinal toxicity. Sci Transl Med.

[25] Rockwell S, Grove TA, Liu Y, Cheng YC, Higgins SA, Booth CJ (2013). Preclinical studies of the Chinese herbal medicine formulation PHY906 (KD018) as a potential adjunct to radiation therapy. Int J Radiat Biol.

[26] Saif MW, Lansigan F, Ruta S, Lamb L, Mezes M, Elligers K (2010). Phase I study of the botanical formulation PHY906 with capecitabine in advanced pancreatic and other gastrointestinal malignancies. Phytomedicine.

[27] Dai J, Liang K, Zhao S, Jia W, Liu Y, Wu H (2018). Chemoproteomics reveals baicalin activates hepatic CPT1 to ameliorate diet-induced obesity and hepatic steatosis. Proc Natl Acad Sci U S A.

[28] Zhang Y, Fox JT, Park YU, Elliott G, Rai G, Cai M (2016). A novel chemotherapeutic agent to treat tumors with DNA mismatch repair deficiencies. Cancer Res.

[29] Li M, Shi A, Pang H, Xue W, Li Y, Cao G (2014). Safety, tolerability, and pharmacokinetics of a single ascending dose of baicalein chewable tablets in healthy subjects. J Ethnopharmacol.

[30] Pang H, Xue W, Shi A, Li M, Li Y, Cao G (2016). Multiple-ascending-dose pharmacokinetics and safety evaluation of baicalein chewable tablets in healthy Chinese volunteers. Clin Drug Investig.

[31] Chiu YW, Lin TH, Huang WS, Teng CY, Liou YS, Kuo WH (2011). Baicalein inhibits the migration and invasive properties of human hepatoma cells. Toxicol Appl Pharmacol.

[32] Patwardhan RS, Sharma D, Thoh M, Checker R, Sandur SK (2016). Baicalein exhibits anti-inflammatory effects via inhibition of NF-kappaB transactivation. Biochem Pharmacol.

[33] Chou CC, Pan SL, Teng CM, Guh JH (2003). Pharmacological evaluation of several major ingredients of Chinese herbal medicines in human hepatoma Hep3B cells. Eur J Pharm Sci.

[34] Huang ST, Lee Y, Gullen EA, Cheng YC (2008). Impacts of baicalein analogs with modification of the 6th position of A ring on the activity toward NF-kappaB-, AP-1-, or CREB-mediated transcription. Bioorg Med Chem Lett.

[35] Samuel T, Fadlalla K, Gales DN, Putcha BD, Manne U (2014). Variable NF-kappaB pathway responses in colon cancer cells treated with chemotherapeutic drugs. BMC Cancer.

[36] Gao J, Wang Y, Xing Q, Yan J, Senthil M, Akmal Y (2011). Identification of a natural compound by cell-based screening that enhances interferon regulatory factor-1 activity and causes tumor suppression. Mol Cancer Ther.

[37] Tian Y, Zhen L, Bai J, Mei Y, Li Z, Lin A (2017). Anticancer effects of baicalein in pancreatic neuroendocrine tumors in vitro and in vivo. Pancreas.

[38] Wang CZ, Zhang CF, Chen L, Anderson S, Lu F, Yuan CS (2015). Colon cancer chemopreventive effects of baicalein, an active enteric microbiome metabolite from baicalin. Int J Oncol.

[39] Srinivas NR (2010). Baicalin, an emerging multi-therapeutic agent: pharmacodynamics, pharmacokinetics, and considerations from drug development perspectives. Xenobiotica.

[40] Di B, Feng N, Liu W (2006). Pharmacokinetic comparisons of Shuang-Huang-Lian with the different combinations of its constitutional herbs. J Ethnopharmacol.

[41] Kim YH, Jeong DW, Kim YC, Sohn DH, Park ES, Lee HS (2007). Pharmacokinetics of baicalein, baicalin and wogonin after oral administration of a standardized extract of Scutellaria baicalensis, PF-2405 in rats. Arch Pharm Res.

[42] Li CR, Zhang L, Wo SK, Zhou LM, Lin G, Zuo Z (2012). Pharmacokinetic interactions among major bioactive components in Radix Scutellariae via metabolic competition. Biopharm Drug Dispos.

[43] Lu T, Song J, Huang F, Deng Y, Xie L, Wang G (2007). Comparative pharmacokinetics of baicalin after oral administration of pure baicalin, Radix scutellariae extract and Huang-Lian-Jie-Du-Tang to rats. J Ethnopharmacol.

[44] Caesar LK, Cech NB (2019). Synergy and antagonism in natural product extracts: when 1 + 1 does not equal 2. Nat Prod Rep.

