# Supplementary material for: Scutellaria baicalensis enhances 5-fluorouracil-based chemotherapy via inhibition of proliferative signaling pathways
Source: Cell Commun Signal. 2023 Jun 19;21:147. doi: 10.1186/s12964-023-01156-7 (PMC10278337; doi:10.1186/s12964-023-01156-7)
Supplement: Supplementary file 2 — Additional file 1: Fig. S1. Effect of HQ on cell cycle distribution.HT-29 cells were treated with HQ for 48 hr, followed by fixation, PI staining, and cell cycle analysis by flow cytometry.The percentage of HT-29 cells in sub G0, G0/G1, S, G2/M phases.The percentage of HCT116, RKO and H630R1 cells in sub G0 phase. Values represent the mean ± S.D. from three independent experiments. **, p < 0.01, versus untreated. Fig. S2. Effect of HQ on signaling pathways.HT29 cells were treated with 0.5 mg/ml HQ for 48 hr and processed for immunoblot analysis.RKO cells stably expressing NF-kB-regulated luciferase were treated with HQ, HQGGT and individual flavonesfor 48 hr. Luciferase activity was determined and normalized to total soluble protein. Fig. S3. Time and dose dependent effects of HQ on RB and TS.HT-29 cells were treated with HQfollowed by processing for immunoblot analysis.HT-29 cells were treated with various concentrations of HQ for 48 hr and processed for immunoblot analysis.HT-29 cells were treated with three different HQ batches for 48 hr, respectively, and processed for immunoblot analysis.HT-29 cells were treated with HQ for 24 hr, then 5-FU was added for another 24 hr and processed for immunoblot analysis. Batch 1: lot#1070831, Batch 2: lot#T2081080, Batch 3: lot#3070715. Fig. S4. Effect of HQ on normal tissues. HQwas orally administered QD × 5 for 6 weeks to MC38-bearing C57BL/6 mice. Formalin-fixed sections of the liver and middle jejunum were stained with hematoxylin and eosin, Ki-67, and TUNEL. Scale bars are 100 μm. Fig. S5. Effect of HQ in combination with DFUR on CRC cell proliferation. MC38 cells were treated with HQ and DFUR for 72 hr. Cell viability was measured by WST-1 assay. The Combination-Indexwas calculated with CI < 1 indicating synergism. Fig. S6. Effect of BI in combination with 5-FU on CRC cell proliferation. RKOR10 cells were treated with various concentrations of BI and 5-FU for 72 hr. Cell viability was measured by WST-1 assay. The Combin [file 12964_2023_1156_MOESM1_ESM.pptx]

## Slide 1
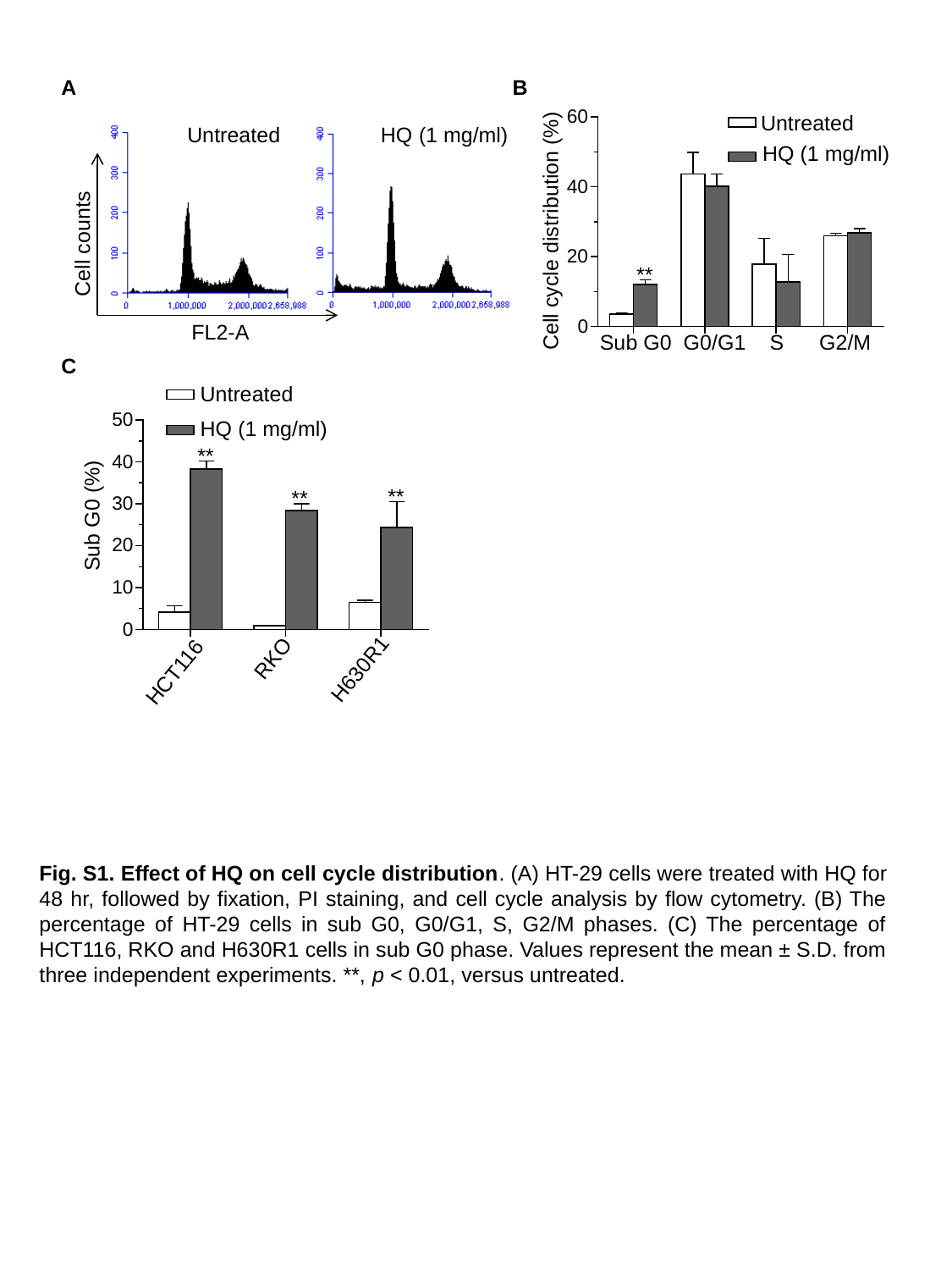

A B
C
Untreated
 Untreated HQ (1 mg/ml)
HQ (1 mg/ml)
Cell cycle distribution (%)
Cell counts
**
FL2-A
 Sub G0 G0/G1 S G2/M
Untreated
HQ (1 mg/ml)
**
**
**
 Sub G0 (%)
H630R1
RKO
HCT116
Fig. S1. Effect of HQ on cell cycle distribution. (A) HT-29 cells were treated with HQ for 48 hr, followed by fixation, PI staining, and cell cycle analysis by flow cytometry. (B) The percentage of HT-29 cells in sub G0, G0/G1, S, G2/M phases. (C) The percentage of HCT116, RKO and H630R1 cells in sub G0 phase. Values represent the mean ± S.D. from three independent experiments. **, p < 0.01, versus untreated.

## Slide 2
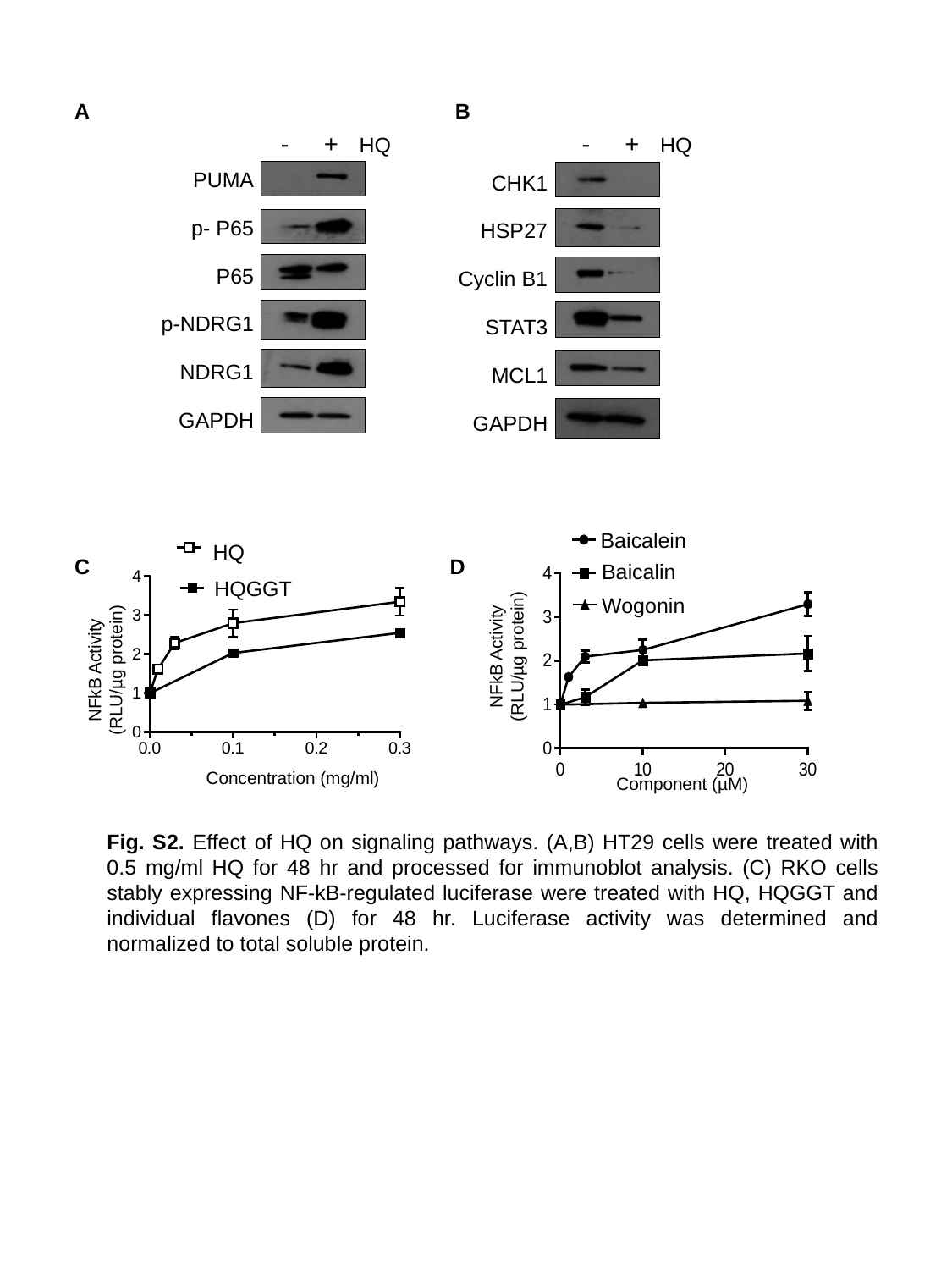

A B
C D
- + HQ - + HQ
PUMA
p- P65
P65
p-NDRG1
NDRG1
GAPDH
CHK1
HSP27
Cyclin B1
STAT3
MCL1
GAPDH
Baicalein
HQ
Baicalin
HQGGT
Wogonin
NFkB Activity
(RLU/µg protein)
NFkB Activity
(RLU/µg protein)
Concentration (mg/ml)
Component (µM)
Fig. S2. Effect of HQ on signaling pathways. (A,B) HT29 cells were treated with 0.5 mg/ml HQ for 48 hr and processed for immunoblot analysis. (C) RKO cells stably expressing NF-kB-regulated luciferase were treated with HQ, HQGGT and individual flavones (D) for 48 hr. Luciferase activity was determined and normalized to total soluble protein.

## Slide 3
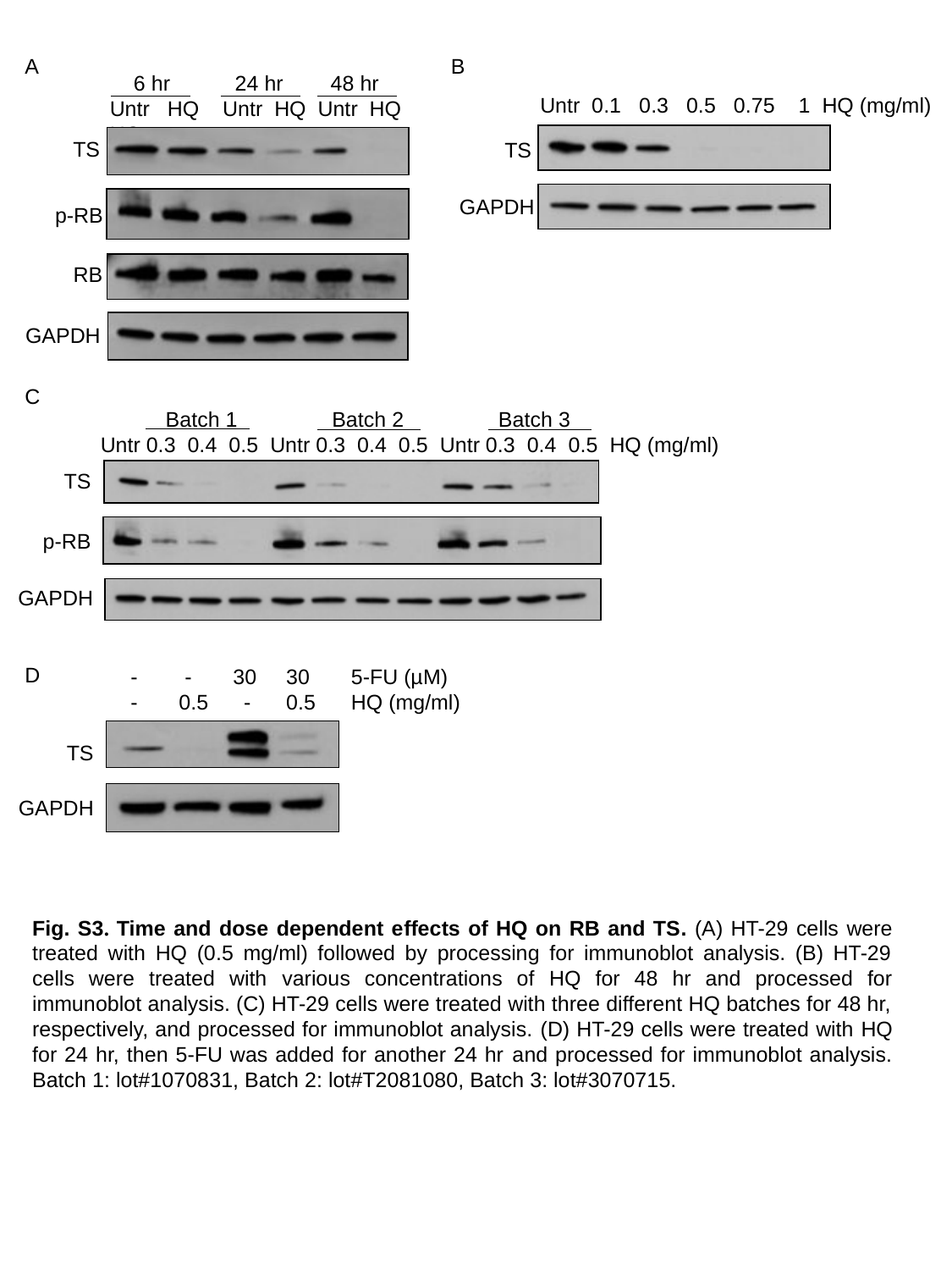

A B
C
D
 6 hr 24 hr 48 hr
Untr HQ Untr HQ Untr HQ HQ
 Untr 0.1 0.3 0.5 0.75 1 HQ (mg/ml)
TS
 TS
 GAPDH
p-RB
RB
GAPDH
 Batch 1 Batch 2 Batch 3
 Untr 0.3 0.4 0.5 Untr 0.3 0.4 0.5 Untr 0.3 0.4 0.5 HQ (mg/ml)
TS
p-RB
GAPDH
 - - 30 30 5-FU (µM)
 - 0.5 - 0.5 HQ (mg/ml)
 TS
GAPDH
Fig. S3. Time and dose dependent effects of HQ on RB and TS. (A) HT-29 cells were treated with HQ (0.5 mg/ml) followed by processing for immunoblot analysis. (B) HT-29 cells were treated with various concentrations of HQ for 48 hr and processed for immunoblot analysis. (C) HT-29 cells were treated with three different HQ batches for 48 hr, respectively, and processed for immunoblot analysis. (D) HT-29 cells were treated with HQ for 24 hr, then 5-FU was added for another 24 hr and processed for immunoblot analysis. Batch 1: lot#1070831, Batch 2: lot#T2081080, Batch 3: lot#3070715.

## Slide 4
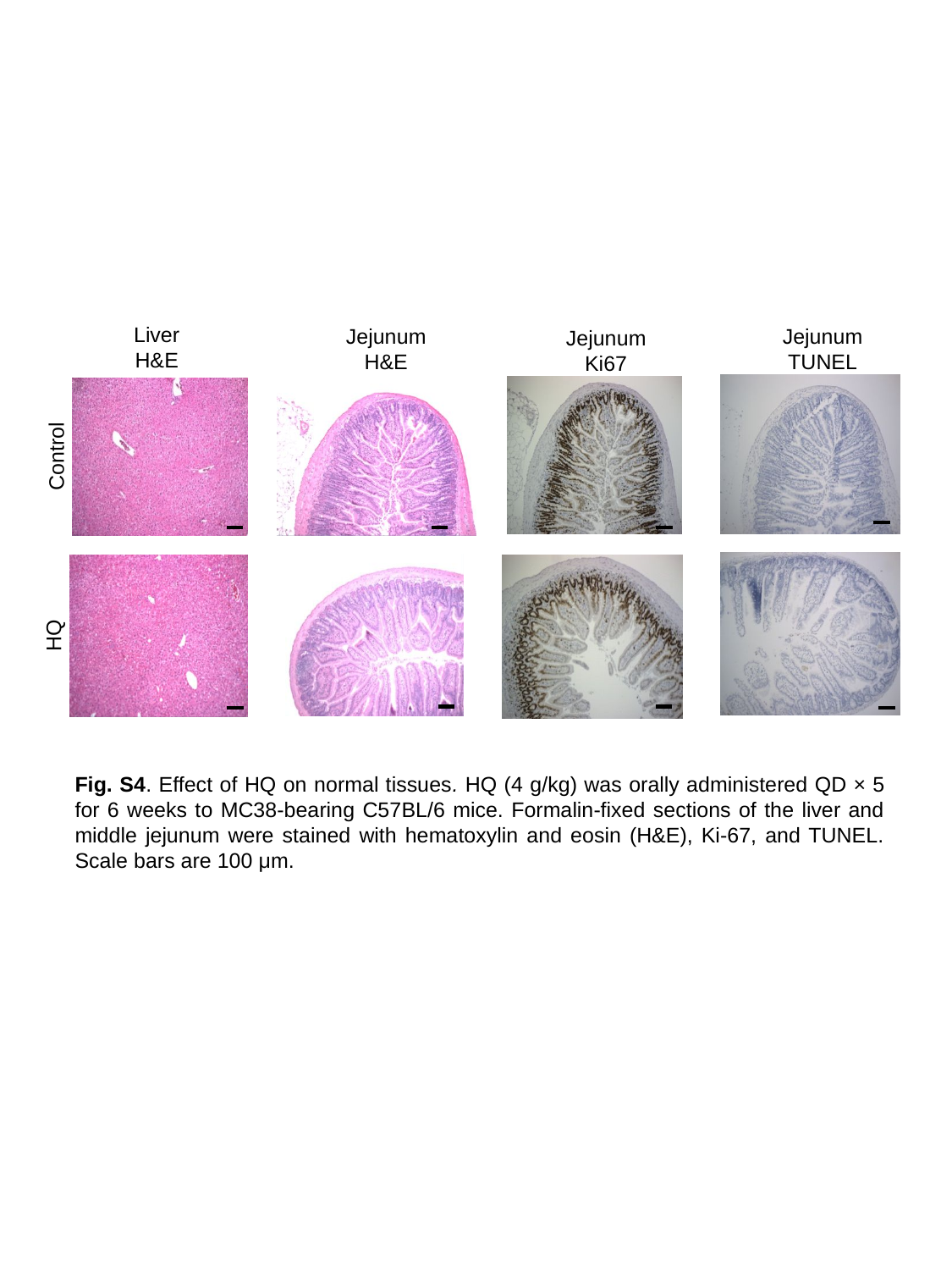

Liver
H&E
Jejunum H&E
Jejunum TUNEL
Jejunum Ki67
Control
HQ
Fig. S4. Effect of HQ on normal tissues. HQ (4 g/kg) was orally administered QD × 5 for 6 weeks to MC38-bearing C57BL/6 mice. Formalin-fixed sections of the liver and middle jejunum were stained with hematoxylin and eosin (H&E), Ki-67, and TUNEL. Scale bars are 100 μm.

## Slide 5
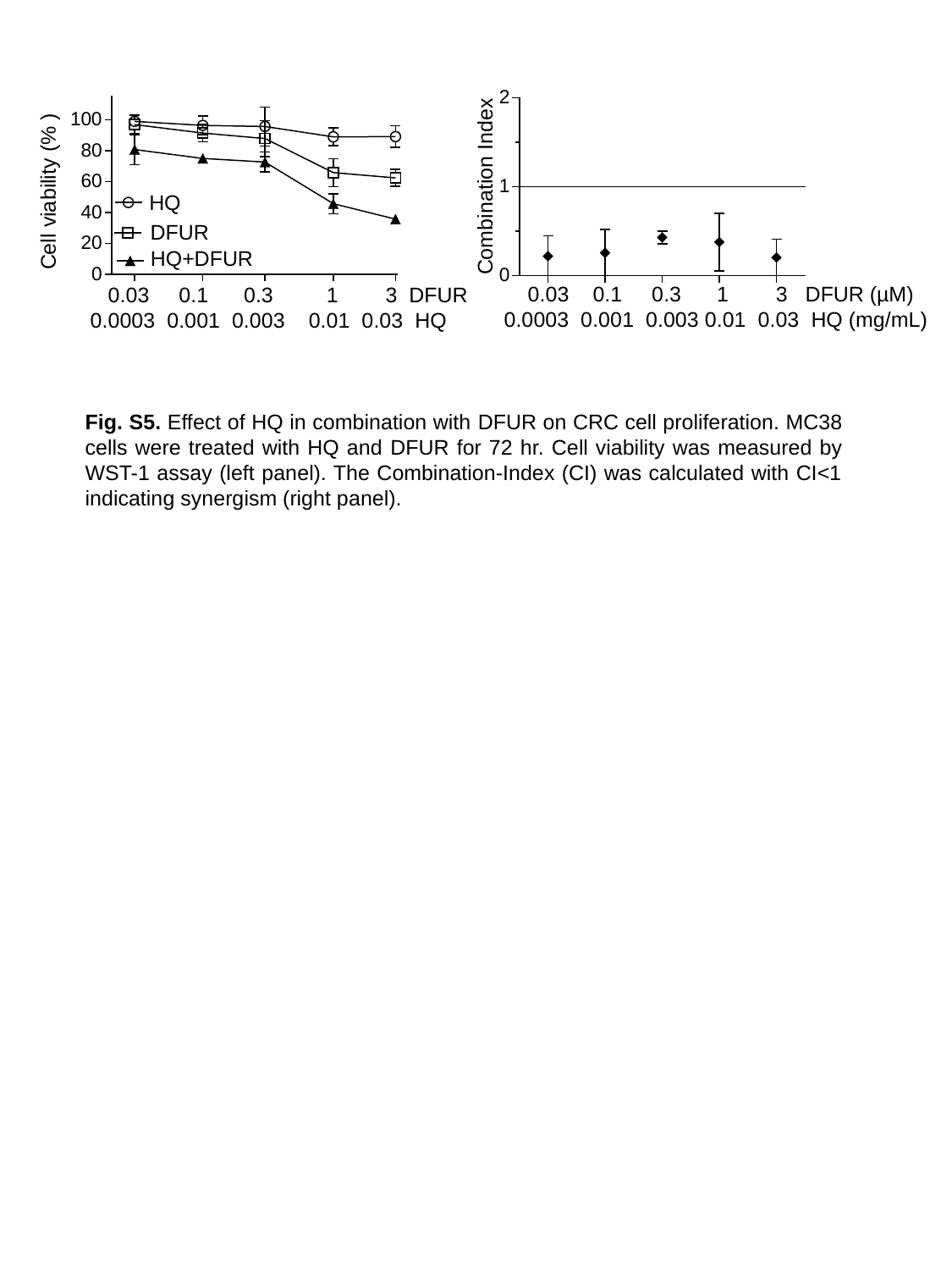

Combination Index
 Cell viability (% )
HQ
DFUR
HQ+DFUR
 0.03 0.1 0.3 1 3 DFUR (µM)
0.0003 0.001 0.003 0.01 0.03 HQ (mg/mL)
 0.03 0.1 0.3 1 3 DFUR
 0.0003 0.001 0.003 0.01 0.03 HQ
Fig. S5. Effect of HQ in combination with DFUR on CRC cell proliferation. MC38 cells were treated with HQ and DFUR for 72 hr. Cell viability was measured by WST-1 assay (left panel). The Combination-Index (CI) was calculated with CI<1 indicating synergism (right panel).

## Slide 6
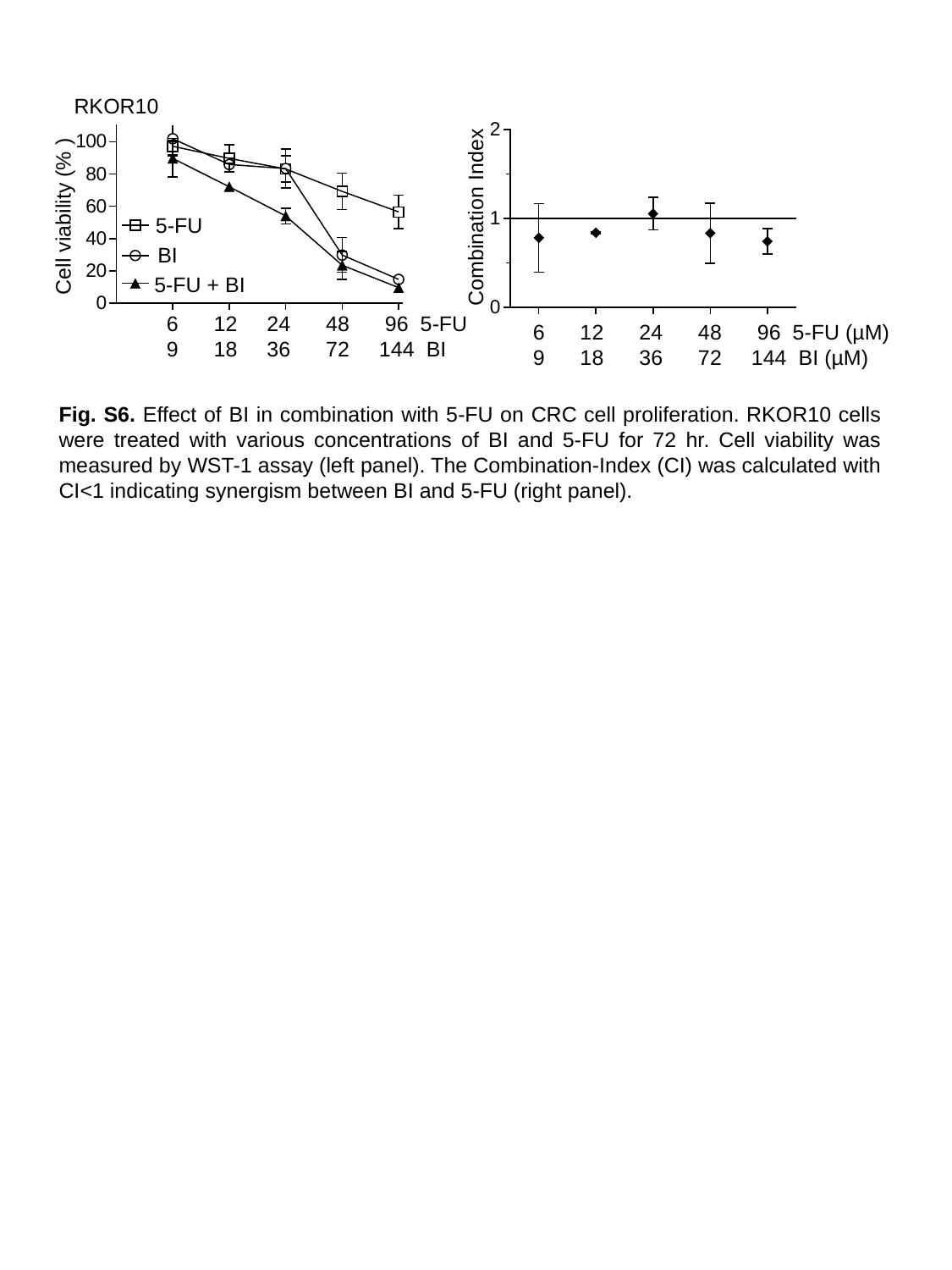

RKOR10
 Cell viability (% )
Combination Index
5-FU
BI
5-FU + BI
 6 12 24 48 96 5-FU
 9 18 36 72 144 BI
 6 12 24 48 96 5-FU (µM)
 9 18 36 72 144 BI (µM)
Fig. S6. Effect of BI in combination with 5-FU on CRC cell proliferation. RKOR10 cells were treated with various concentrations of BI and 5-FU for 72 hr. Cell viability was measured by WST-1 assay (left panel). The Combination-Index (CI) was calculated with CI<1 indicating synergism between BI and 5-FU (right panel).

## Slide 7
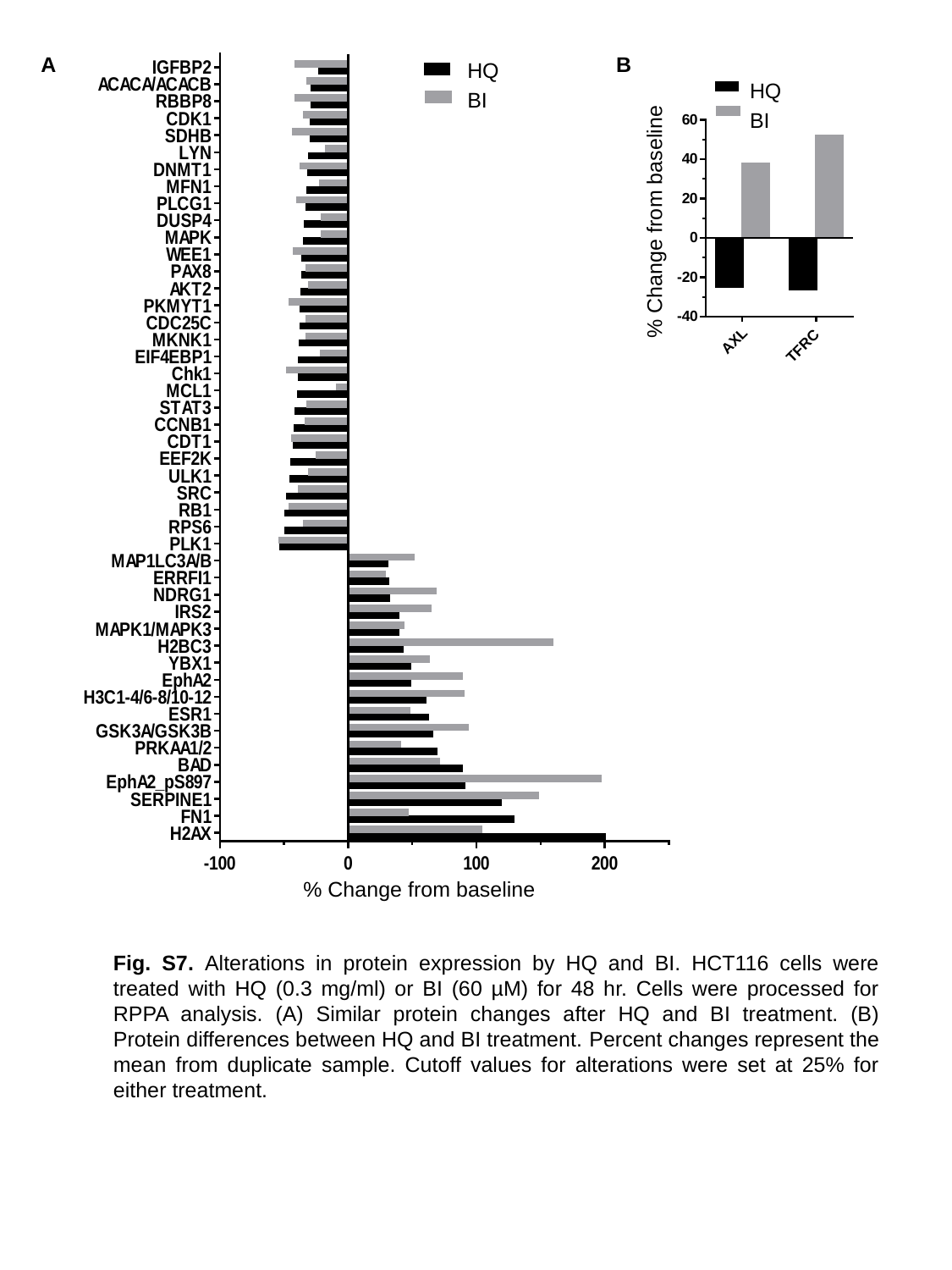

A B
HQ
BI
HQ
BI
 % Change from baseline
 % Change from baseline
Fig. S7. Alterations in protein expression by HQ and BI. HCT116 cells were treated with HQ (0.3 mg/ml) or BI (60 µM) for 48 hr. Cells were processed for RPPA analysis. (A) Similar protein changes after HQ and BI treatment. (B) Protein differences between HQ and BI treatment. Percent changes represent the mean from duplicate sample. Cutoff values for alterations were set at 25% for either treatment.

## Slide 8
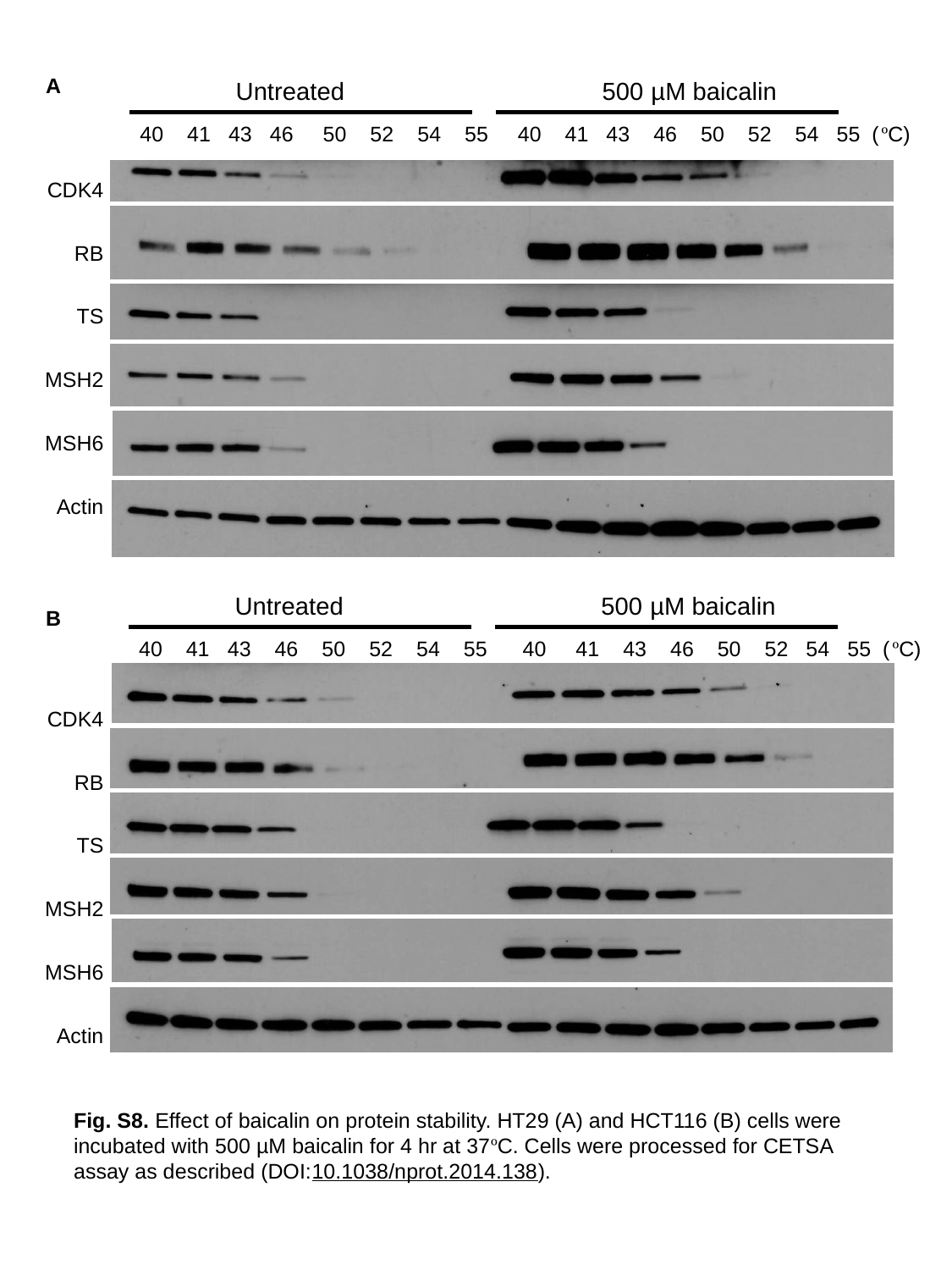

A
B
Untreated 500 µM baicalin
40 41 43 46 50 52 54 55 40 41 43 46 50 52 54 55 (ºC)
CDK4
RB
TS
MSH2
MSH6
Actin
Untreated 500 µM baicalin
40 41 43 46 50 52 54 55 40 41 43 46 50 52 54 55 (ºC)
CDK4
RB
TS
MSH2
MSH6
Actin
Fig. S8. Effect of baicalin on protein stability. HT29 (A) and HCT116 (B) cells were incubated with 500 µM baicalin for 4 hr at 37ºC. Cells were processed for CETSA assay as described (DOI:10.1038/nprot.2014.138).
