# Supplementary material for: Scutellaria baicalensis enhances 5-fluorouracil-based chemotherapy via inhibition of proliferative signaling pathways
Source: Cell Commun Signal. 2023 Jun 19;21:147. doi: 10.1186/s12964-023-01156-7 (PMC10278337; doi:10.1186/s12964-023-01156-7)
Supplement: Supplementary file 3 — Additional file 2: Table S1. HQ IC50 values in CRC cells. Table S2. IC50 values of HQ against colon cancer cell lines. Table S3. Flavone concentration in three different HQ batches. Table S4. Proteins altered following HQ treatment. Table S5. Flavone IC50 values in HT-29 cells. [file 12964_2023_1156_MOESM2_ESM.pptx]

## Slide 1
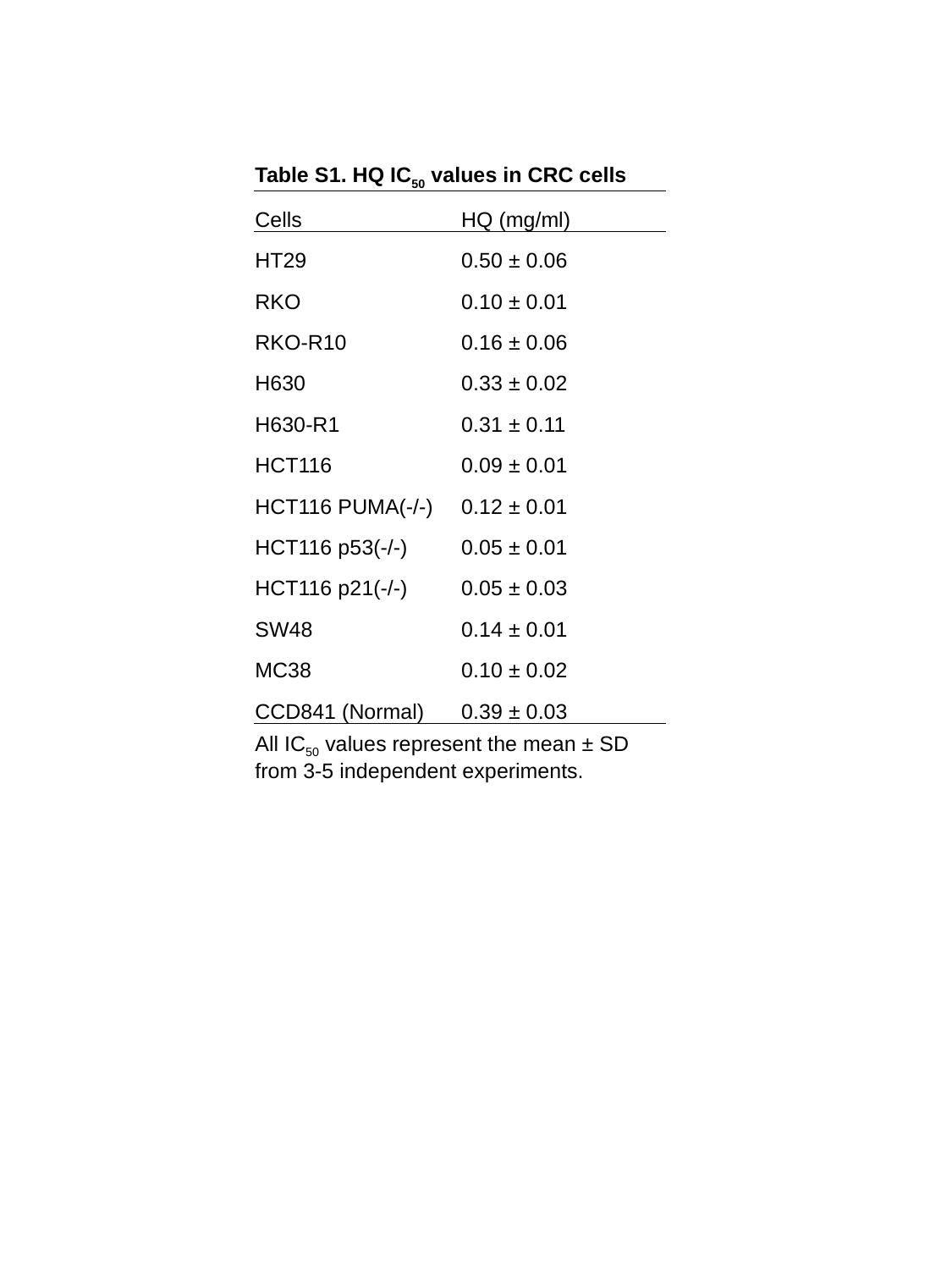

| Table S1. HQ IC50 values in CRC cells | |
| --- | --- |
| Cells | HQ (mg/ml) |
| HT29 | 0.50 ± 0.06 |
| RKO | 0.10 ± 0.01 |
| RKO-R10 | 0.16 ± 0.06 |
| H630 | 0.33 ± 0.02 |
| H630-R1 | 0.31 ± 0.11 |
| HCT116 | 0.09 ± 0.01 |
| HCT116 PUMA(-/-) | 0.12 ± 0.01 |
| HCT116 p53(-/-) | 0.05 ± 0.01 |
| HCT116 p21(-/-) | 0.05 ± 0.03 |
| SW48 | 0.14 ± 0.01 |
| MC38 | 0.10 ± 0.02 |
| CCD841 (Normal) | 0.39 ± 0.03 |
| All IC50 values represent the mean ± SD from 3-5 independent experiments. | |

## Slide 2
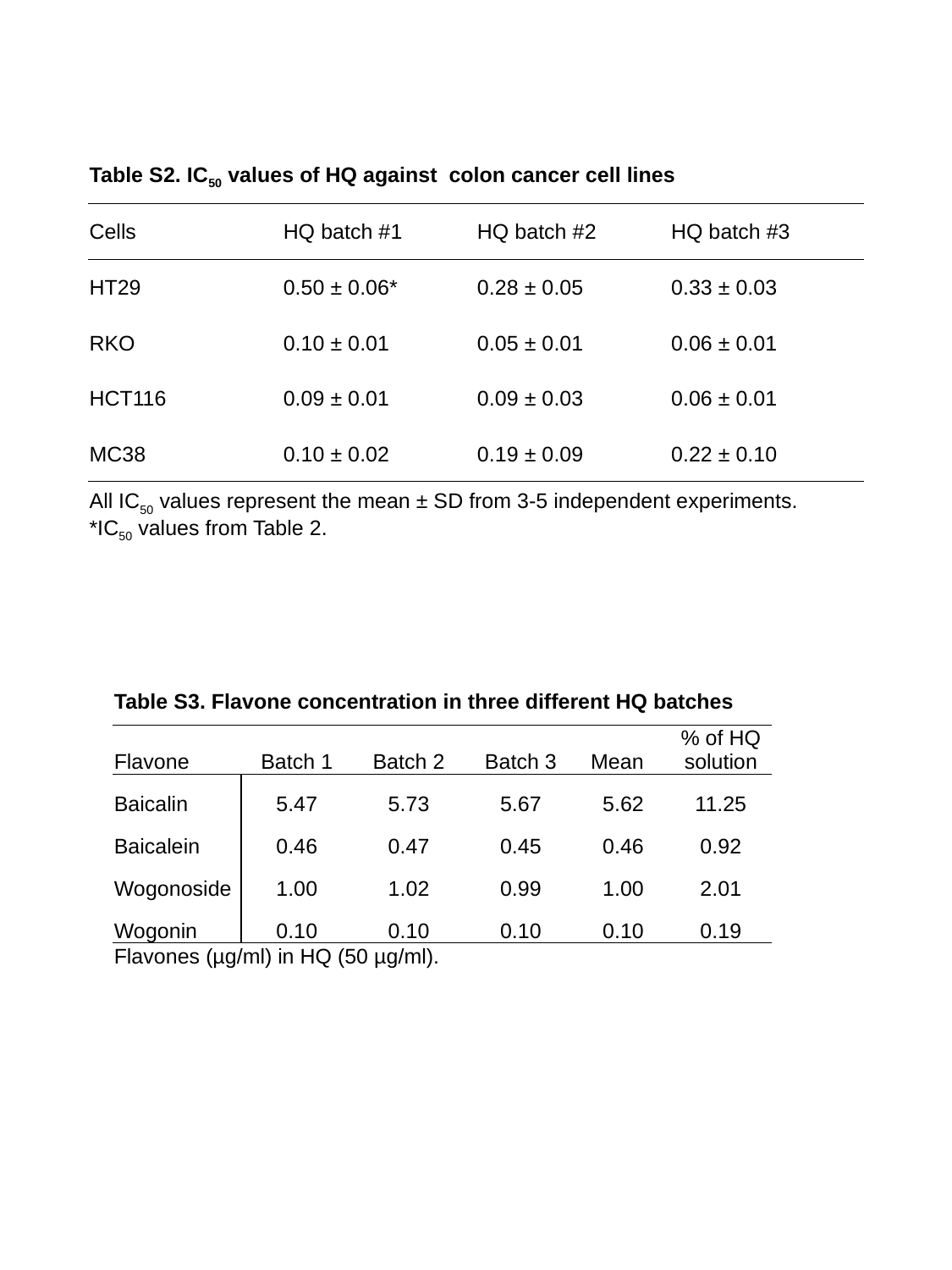

| Table S2. IC50 values of HQ against colon cancer cell lines | | | |
| --- | --- | --- | --- |
| Cells | HQ batch #1 | HQ batch #2 | HQ batch #3 |
| HT29 | 0.50 ± 0.06\* | 0.28 ± 0.05 | 0.33 ± 0.03 |
| RKO | 0.10 ± 0.01 | 0.05 ± 0.01 | 0.06 ± 0.01 |
| HCT116 | 0.09 ± 0.01 | 0.09 ± 0.03 | 0.06 ± 0.01 |
| MC38 | 0.10 ± 0.02 | 0.19 ± 0.09 | 0.22 ± 0.10 |
| All IC50 values represent the mean ± SD from 3-5 independent experiments. \*IC50 values from Table 2. | | | |
| Table S3. Flavone concentration in three different HQ batches | | | | | |
| --- | --- | --- | --- | --- | --- |
| Flavone | Batch 1 | Batch 2 | Batch 3 | Mean | % of HQ solution |
| Baicalin | 5.47 | 5.73 | 5.67 | 5.62 | 11.25 |
| Baicalein | 0.46 | 0.47 | 0.45 | 0.46 | 0.92 |
| Wogonoside | 1.00 | 1.02 | 0.99 | 1.00 | 2.01 |
| Wogonin | 0.10 | 0.10 | 0.10 | 0.10 | 0.19 |
| Flavones (µg/ml) in HQ (50 µg/ml). | | | | | |

## Slide 3
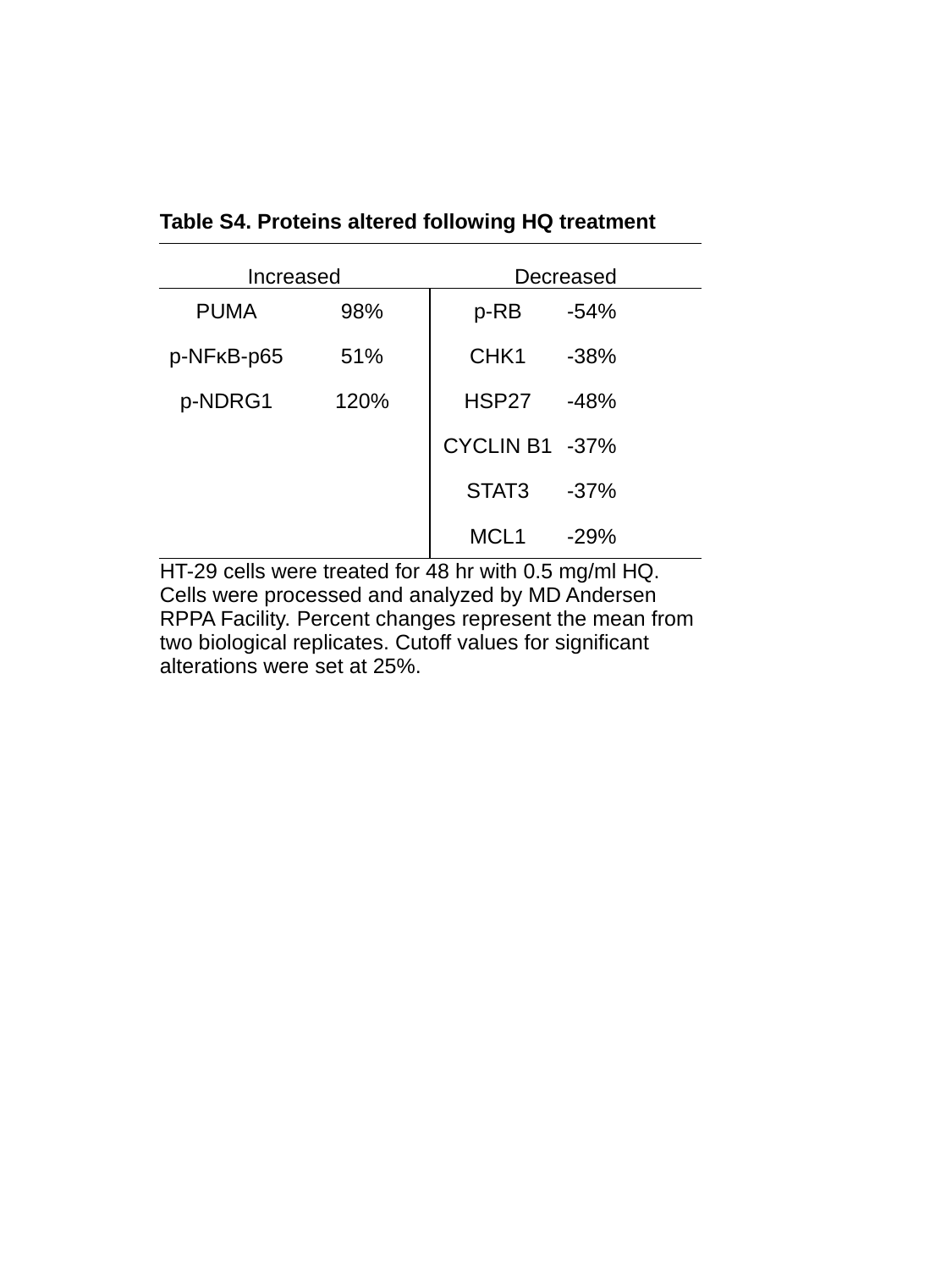

| Table S4. Proteins altered following HQ treatment | | | |
| --- | --- | --- | --- |
| Increased | | Decreased | |
| PUMA | 98% | p-RB | -54% |
| p-NFκB-p65 | 51% | CHK1 | -38% |
| p-NDRG1 | 120% | HSP27 | -48% |
| | | CYCLIN B1 | -37% |
| | | STAT3 | -37% |
| | | MCL1 | -29% |
| HT-29 cells were treated for 48 hr with 0.5 mg/ml HQ. Cells were processed and analyzed by MD Andersen RPPA Facility. Percent changes represent the mean from two biological replicates. Cutoff values for significant alterations were set at 25%. | | | |

## Slide 4
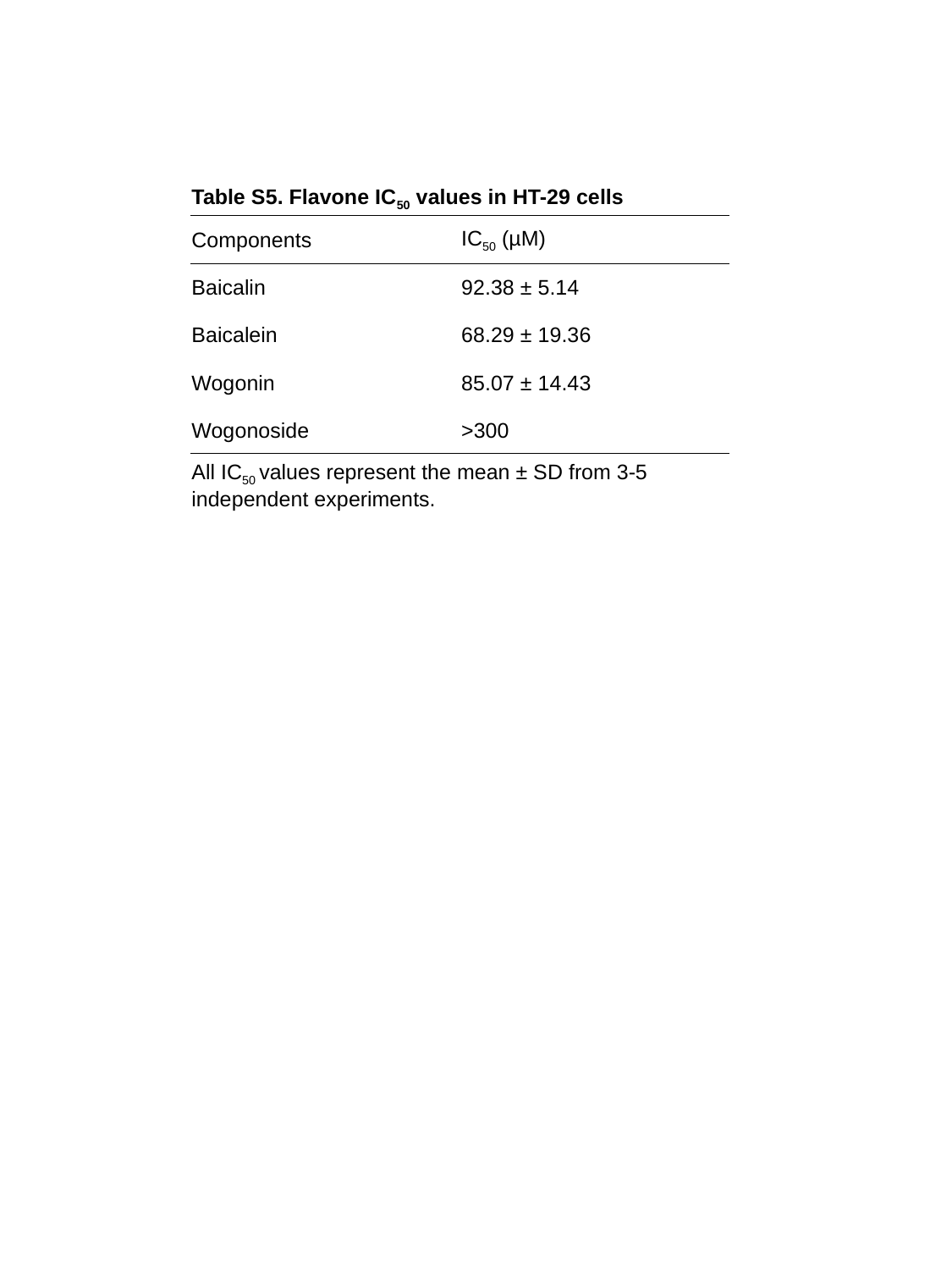

| Table S5. Flavone IC50 values in HT-29 cells | |
| --- | --- |
| Components | IC50 (µM) |
| Baicalin | 92.38 ± 5.14 |
| Baicalein | 68.29 ± 19.36 |
| Wogonin | 85.07 ± 14.43 |
| Wogonoside | >300 |
| All IC50 values represent the mean ± SD from 3-5 independent experiments. | |
